# Enhanced sensory, antioxidant, and non-toxic anti-diabetes beverage development via co-culture fermentation of *Lactiplantibacillus plantarum* and *Saccharomyces boulardii* in coffee cherry pulp extracts

**DOI:** 10.1016/j.fochx.2026.103524

**Published:** 2026-01-12

**Authors:** Supanut Pothimoi, Phisit Seesuriyachan, Thanongsak Chaiyaso, Chayatip Insomphun, Kamon Yakul, Yuthana Phimolsiripol, Churairat Moukamnerd

**Affiliations:** a School of Agro-Industry, Faculty of Agro-Industry, Chiang Mai University, Chiang Mai 50100, Thailand; b Interdisciplinary Program in Biotechnology, Multidisciplinary and Interdisciplinary School, Chiang Mai University, Chiang Mai 50200, Thailand; c Advanced Technology and Innovation Management for Creative Economy Research Group (AIMCE), Department of Industrial Engineering, Faculty of Engineering, Chiang Mai University, Chiang Mai 50200, Thailand

**Keywords:** *Lactiplantibacillus plantarum*, *Saccharomyces boulardii*, Co-culture fermentation, Antioxidant activity, Bioactive compounds, Functional beverage

## Abstract

Coffee cherry pulp (CC), a phenolic-rich by-product of coffee processing, is often discarded, raising sustainability concerns. This study investigated co-culture fermentation of extracted CC using *Lactiplantibacillus plantarum* TISTR 2070 and *Saccharomyces boulardii* CNCM I-745 to enhance its functional potential. Conditions optimized via Box–Behnken design (7.30 log CFU mL^−1^ LAB, 2.52 log CFU mL^−1^ yeast, 30 °C, and 26 h) led to increases of 19% in total phenolic content and 26% in DPPH activity. Potent inhibition of α-amylase and α-glucosidase (*IC*_*50*_: 4.67 and 3.13 mg mL^−1^, respectively) suggests efficacy against carbohydrate-hydrolyzing enzymes. Cytotoxicity tests confirmed safety up to 500 μg mL^−1^ in Vero cells. Ethanol content remained low (1.40% *v*/v ABV), aligning with the characteristics of low-alcohol fermented beverages, the beverage exhibited favorable sensory acceptability (7.5/9 from 50 panelists), This strategy enhances resource utilization, supports circular food systems, and advances the Sustainable Development Goals.

## Highlights

• Novel co-culture fermentation of upcycles coffee cherry pulp into a functional beverage.
• Optimized LAB–yeast synergy boosted phenolics by 19% and antioxidants by 26%.
• Fermented extract strongly inhibits both α-amylase and α-glucosidase.
• Non-toxic and sensory accepted, supporting future commercial development.
• Promotes sustainable valorization of agro-waste via bioprocessing innovation.

## Graphical abstract

**Figure:**
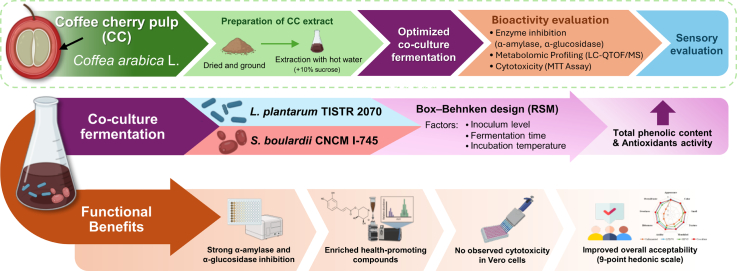

## 1 Introduction

Coffee is one of the most widely consumed beverages and represents a major traded commodity worldwide. According to the USDA Foreign Agricultural Service (2025), global coffee production in 2025/26 is projected to reach a record 10.72 million metric tons, while consumption is expected to total 10.16 million metric tons, underscoring strong global demand. Nevertheless, coffee production generates substantial quantities of agricultural by-products, with approximately 43–50% of coffee cherry pulp derived from wet processing (Tsigkou et al., 2025). If improperly managed, these residues can pose serious environmental threats. Their disposal into water bodies and landfills without pretreatment can deteriorate water quality, harm local ecosystems (Pandey et al., 2025).

The utilization of coffee cherry pulp as animal feed (Chaiyaso et al., 2025) is a prevalent strategy to address environmental issues. Consequently, a growing interest in the circular bioeconomy has notably heightened the enhancement of coffee cherry pulp for human consumption (Tsigkou et al., 2025).

In 2022, the European Food Safety Authority (EFSA) identified coffee cherry pulp as a novel food in the EU market (EFSA Panel on Nutrition, Novel Foods and Food Allergens, 2022). Rich in proteins, carbohydrates, lipids, minerals, and especially phenolic compounds, coffee cherry pulp is increasingly recognized as a promising sustainable and functional food ingredient.

Fermentation is an inexpensive, widely used method for improving food preservation and producing bioactive compounds, making fermented products appealing for their health benefits (Morales-de la Peña et al., 2023). For plant-based substrates like coffee cherry pulp, fermentation can boost the bioavailability and bioactivity of compounds through microbial metabolism, which is affected by the raw material composition and microbial selection.

Although single-culture fermentation with lactic acid bacteria (LAB) has been shown to enhance phenolic content and antioxidant activity in coffee cherry pulp (Myo et al., 2021), co-culture fermentation using LAB and yeast in this specific, complex waste matrix remains largely unexplored in a systematic manner. Evidence from other food and by-product matrices supports the high potential of LAB–yeast co-culture systems. For example, co-culture fermentation of mature coconut water and green tea has been reported to improve sensory attributes, enhance microbial viability, and increase overall product complexity compared to single cultures (Wang et al., 2022; Xu et al., 2025). These effects are primarily attributed to synergistic microbial interactions, in which LAB and yeasts cooperatively transform bound phenolic compounds into more bioactive forms through complementary enzymatic activities, such as esterases and β-glucosidases (Yuan et al., 2025).

Previous studies from our group established the feasibility of valorizing coffee cherry pulp and enhancing its bioactive potential through fermentation (Chomphoosee et al., 2025; Moukamnerd et al., 2025). Building on these findings, the present study advances this research by investigating a defined LAB–yeast co-culture system and systematically optimizing fermentation parameters using Response Surface Methodology (RSM).

The novelty of this work lies in applying RSM to a specific co-culture of *Lactiplantibacillus plantarum* TISTR 2070 and *Saccharomyces boulardii* CNCM I-745, enabling the transition from exploratory fermentation studies toward reproducible and scalable processing conditions. Crucially, this study advances functional beverage research beyond existing co-culture fermentations in conventional matrices by combining the sustainable valorization of coffee cherry pulp agricultural waste with this robust optimization framework.

Therefore, this study aims to optimize co-culture fermentation parameters (LAB–yeast inoculum ratio, fermentation time, and temperature) using Box–Behnken Design and Response Surface Methodology (RSM) to enhance total phenolic content and antioxidant capacity. The fermented coffee cherry pulp extract was further evaluated for its inhibitory effects on diabetes-related enzymes, cytotoxicity, and sensory acceptability, to assess its potential as safe, bioactive-rich, and functional food ingredients.

## 2 Materials and methods

### 2.1 Raw material

Coffee cherry pulp (CC) was obtained from *Coffea arabica* L. grown at a coffee processing facility in Thep Sadet Subdistrict, Doi Saket District, Chiang Mai Province, Thailand. The pulp was thoroughly washed with distilled water to remove pulp and dried in a hot-air oven at 60 °C until it reached a constant weight. The dried pulp was ground into a fine powder using a laboratory blender and sifted through an 18-mesh sieve. Then, it was vacuum-packed and stored at 25 °C.

### 2.2 Preparation of CC extract

The CC powder was extracted in hot water at a concentration of 100 g L^−1^ at 90 °C for 15 min under continuous stirring. Sucrose was then added at a concentration of 100 g L^−1^. The mixture was thoroughly homogenized and subsequently filtered through muslin cloth to remove insoluble residues.

### 2.3 Microbial strain preparation

The microbial strains used in this study included *Saccharomyces boulardii* CNCM I-745 (SB745) and *Lactiplantibacillus plantarum* 299v (LP299V), both of which were isolated from a commercially available probiotic product and sub-cultured for laboratory use, as well as *Saccharomyces cerevisiae* TISTR 6034 (SC6034) and *Lactiplantibacillus plantarum* TISTR 2070 (LP2070), obtained from the Thailand Institute of Scientific and Technological Research (TISTR, Thailand). Yeast strains (SB745 and SC6034) were cultivated in yeast malt extract (YM) medium (HiMedia, India) at 30 °C for 24 h, while lactic acid bacteria (LP299V and LP2070) were cultured in de Man, Rogosa and Sharpe (MRS) medium (HiMedia, India) at 30 °C under static conditions for 24 h.

After incubation, the optical density at 600 nm (OD₆₀₀) was adjusted to 0.8–1.0, corresponding to 6–8 log CFU mL^−1^, based on a previously established correlation between OD₆₀₀ and viable cell counts (Moukamnerd et al., 2025). Cells were then harvested by centrifugation at 6000 ×*g* for 15 min at 4 °C, washed twice with sterile 0.02% (*w*/*v*) NaCl solution, and resuspended in the same solution. The resulting suspensions were freshly prepared and immediately used in subsequent fermentation experiments.

All microbial strains were handled separately in laboratory conditions and used exclusively for non-commercial academic research. No endorsement or affiliation with the producers of the probiotic products is implied.

### 2.4 Fermentations

#### 2.4.1 Single-culture fermentations

The CC extract was inoculated with individual microbial strains to achieve an initial microbial load of approximately 6 log CFU mL^−1^. Four strains were employed in this experiment: *S. boulardii* CNCM I-745 (SB745), *S. cerevisiae* TISTR 6034 (SC6034), *L. plantarum* 299v (LP299V), and *L.* *plantarum* TISTR 2070 (LP2070). The inoculated samples were incubated at 30 °C for 72 h. Fermentation samples were collected every 24 h to evaluate biological and physicochemical properties further, as described in Section 2.5. Strains exhibiting the highest total phenolic content (TPC) and antioxidant activities (DPPH, ABTS, and FRAP assays) were selected as candidates for subsequent co-culture fermentation.

#### 2.4.2 Co-culture fermentations

Co-culture fermentation was optimized using a Box–Behnken design (BBD) under Response Surface Methodology (RSM), considering four independent variables: the initial inoculum levels of LAB and yeast strains (log CFU mL^−1^), fermentation time (h) and temperature (°C). Based on preliminary screening results for bioactivity, *L. plantarum* TISTR 2070 and *S. boulardii* CNCM I-745 were selected as the co-culture pair for optimization. The individual starter cultures were prepared similarly to the single-culture fermentations (Section 2.4.1), but their initial inoculum levels were adjusted according to the specific design of the Box–Behnken design.

The responses measured were total phenolic content, and antioxidant activity (DPPH, ABTS, and FRAP assays). Table 1 presents the coded values for the four parameters utilized in this study. The minimum code value was −1, the median value was 0, and the maximum value was +1. The design consisted of 29 experimental runs, including five replicates at the center point, to allow reliable estimation of pure error and to validate the adequacy of the model.

**Table 1:** Factors and levels of the Box–Behnken experiment design.

Factor		Level		
		**-1**	**0**	**+1**
LAB strain, log CFU mL^−1^	A	6	7	8
Yeast strain, log CFU mL^−1^	B	0	2	4
Time, h	C	0	24	48
Temperature, °C	D	25	30	35

The Box–Behnken experimental design employed four independent variables, with ranges selected to capture an optimal operational window for LAB–yeast co-culture fermentation. LAB inoculum levels (6–8 log CFU mL^−1^) were chosen to ensure sufficient dominance (>7 log CFU mL^−1^) required for effective enzymatic biotransformation of phenolic compounds and sustained functional activity (Chan et al., 2021). In contrast, yeast inoculum levels were set lower (0–4 log CFU mL^−1^) to moderate yeast metabolism, facilitate rapid LAB-driven acidification, and reduce the risk of excessive yeast growth and off-flavor formation (Nyhan et al., 2023).

The temperature range (25–35 °C) was selected to encompass the physiological activity range of both microorganisms, including the previously reported optimal temperature (30 °C) for *L.* *plantarum* fermentation of coffee pulp (Myo et al., 2021). Fermentation time was limited to 0–48 h to capture the early metabolic phase characterized by rapid acidification and initial metabolite accumulation (Dysvik et al., 2020). All experimental runs were conducted in a fully randomized order to minimize systematic bias and ensure statistical robustness.

### 2.5 Analysis of biological and physicochemical properties

#### 2.5.1 Quantification of viable cells

The determination of lactic acid bacteria (LAB) and yeasts in CC extract fermentation was conducted by the spread plate technique on designated culture media. LAB was quantified on de Man, Rogosa and Sharpe (MRS) agar augmented with nystatin 100 IU mL^−1^ (Stodolak et al., 2024) and incubated for 24–48 h until distinct colonies were observed for enumeration.

Yeasts were quantified on potato dextrose agar (PDA) augmented with 1% (*v*/v) sterilized solution of 10% tartaric acid to suppress bacterial proliferation and incubated at 30 °C for 24 h. Isolated colonies were enumerated and reported as log colony-forming units (CFU mL^−1^).

#### 2.5.2 Examination of pH, sugar, ethanol and lactic acid

The pH of CC extract fermentation was assessed using an electronic pH meter (10BNC, Singapore). Quantification of sugars (sucrose, glucose, and fructose), lactic acid, and ethanol was performed using high-performance liquid chromatography (HPLC) system (Hitachi Chromaster 5450, Japan) equipped with a Refractive Index Detector (RID) and an Aminex HPX-87H column (7.8 × 300 mm, 10 μm). The mobile phase was 5 mM H₂SO₄ at a constant flow rate of 0.4 mL min^−1^ with the column maintained at 45 °C. Samples were filtered through 0.22 μm nylon syringe filters prior to analysis, and data were processed using ChromAssist (version 2.1). The outcomes are articulated in g L^−1^ (Panti et al., 2024).

#### 2.5.3 Total phenolic content

The total phenolic content during CC extract fermentation was assessed using the Folin-Ciocalteu technique (Geremu et al., 2016). The measurement is expressed as micrograms of gallic acid equivalents per milliliter (μg GAE mL^−1^), based on a calibration curve constructed with standard solutions of gallic acid.

#### 2.5.4 Antioxidant capacity

The antioxidant activity was assessed by three assays: DPPH, ABTS, and FRAP. The free radical scavenging activity of 2,2-diphenyl-1-picrylhydrazyl (DPPH) was assessed by combining 500 μL of the sample solution with 1000 μL of DPPH solution, followed by gentle shaking and incubation in darkness at 30 °C for 30 min prior to measurement of absorbance at 517 nm (Xia et al., 2014). The free radical scavenging test utilizing 2,2′-azino-bis (3-ethylbenzothiazoline-6-sulfonic acid) (ABTS) was using 1000 μL of the produced ABTS working solution with 50 μL of the sample solution. The liquids were thereafter combined and incubated in a dark place for 7 min prior to measuring the absorbance at 734 nm (Xiao et al., 2020). The reducing potential of the samples was assessed by the Ferric Reducing Antioxidant Power (FRAP) assay by combining 900 μL of FRAP reagent solution with 25 μL of sample solution, incubating in darkness at 37 °C for 15 min, and measuring the absorbance at 593 nm (Xiao et al., 2020). All test findings were quantified as Trolox equivalent (TE) antioxidants and shown in μg TE mL^−1^.

### 2.6 Alpha-amylase and alpha-glucosidase inhibition

Enzyme inhibitory activities were assessed using co-culture fermented CC extract obtained under optimized conditions and compared with those from single-culture fermentations performed under the same conditions.

The α-amylase assay for carbohydrate hydrolysis was conducted using the approach given by Alyahya et al. (2021). Alpha-amylase enzyme from porcine pancreas, and a 0.5% starch solution served as the substrate. The assay combination included 150 μL of α-amylase enzyme solution and 150 μL of fermented CC at different concentrations (0.05, 0.5, 5.0, 7.5, and 10 mg mL^−1^) and incubated at 37 °C for 10 min. Then, 150 μL of a 5 g L^−1^ starch solution was added to initiate the reaction, and the tubes were incubated at 37 °C for 30 min. Subsequently, 300 μL of 3,5-dinitrosalicylic acid (DNS) was added to halt the reaction, and the tubes were incubated in a water bath at 100 °C for 10 min. Ultimately, all tubes were equilibrated to room temperature and then diluted with 2 mL of distilled water. Absorbance was quantified at 540 nm using a TECAN Spark Multimode Microplate Reader (TECAN, United States).

The α-glucosidase inhibition assay was adapted from a method from Patil et al. (2022). Alpha-glucosidase was produced by *S. cerevisiae*, and *p*-nitrophenyl-α-D-glucoside (*p*NPG) was employed as a substrate. In the assay, CC fermented was prepared at different concentrations (0.5, 2.5, 5.0, 7.5, and 10 mg mL^−1^) with a volume of 40 μL and 40 μL of 0.05 M sodium phosphate buffer (pH 6.8) was added. Thereafter adding 50 μL of α-glucosidase enzyme (1 unit mL^−1^), the mixture was incubated at 37 °C for 15 min. After preincubation, 50 μL of 3 mM *p*NPG solution was added to all wells. Then, incubate the reaction mixture at 37 °C for 20 min. The reaction was stopped by adding 50 μL of sodium carbonate. The activity of the extract was assessed spectrophotometrically by measuring the yellow *p*-nitrophenol generated from *p*NPG at 405 nm, utilizing a TECAN Spark Multimode Microplate Reader (TECAN, United States).

The inhibitory activities of both α-amylase and α-glucosidase were quantified as *IC*_*50*_, derived from concentration-inhibition calibration curves consisting of five calibration points. Acarbose (5 mg mL^−1^), a commercially available inhibitor, served as the positive control. The inhibitory activity percentage of each sample was assessed using the following equations:

$$\mathrm{Inhibition}\;\left(\%\right)=\frac{A_{Control}-\left(A_{Sample}-A_{Control\;Sample}\right)}{A_{Control}}x\;100$$

### 2.7 LC-QTOF/MS analysis

Metabolite profiling was performed using an Agilent 1290 Infinity II UHPLC system coupled with an Agilent 6545B QTOF/MS via a Dual AJS electrospray ionization (ESI) source. Separation was achieved on an Agilent Poroshell 120 EC-C18 column (2.1 × 150 mm, 2.7 μm) with a mobile phase of 0.1% (*v*/v) formic acid in water (A) and 0.1% (v/v) formic acid in acetonitrile (B), at a flow rate of 0.2 mL min^−1^. The gradient was programmed as follows: 5% B (0–1 min), increased to 17% B (10–13 min), to 95% B (20–25 min), and then returned to 5% B by 27 min, re-equilibrated until 35 min. The column temperature was set at 35 °C, and the injection volume was 1 μL.

MS detection was operated in both positive and negative ionization modes (*m/z* 100–1100) at 2 spectra s^−1^. The source parameters were drying gas 300 °C (10 L min^−1^), sheath gas 350 °C (11 L min^−1^), nebulizer 35 psig, capillary voltage 3500 V, nozzle voltage 1000 V, fragmentor 175 V, skimmer 65 V, and octopole RF 750 V. Continuous mass calibration was performed using reference ions at *m/z* 121.0509 and 922.0098 for positive mode, and *m/z* 112.9855 and 966.0007 for negative mode. The method accuracy and stability were confirmed internally via this continuous mass calibration. Raw data were acquired and processed using Agilent MassHunter software suite (Data Acquisition B.08.00; Qualitative Analysis B.08.00). Compound identification was performed via the Find by Molecular Feature (MFE) algorithm to extract metabolic features. The identification of metabolites was achieved through a Database Search (DBSearch) against the automated library. Metabolite identification was validated based on accurate mass and isotopic distribution, maintaining a mass tolerance of ≤5 ppm throughout the analysis.

### 2.8 Cytotoxicity test

Cytotoxicity of the CC extract fermentation was assessed using the MTT assay, with slight modifications from the method described by Rattanaburee et al. (2022). Vero cells (African green monkey kidney epithelial cells; ATCC® CCL-81™) were selected in this preliminary cytotoxicity evaluation due to their established sensitivity and robustness in general mammalian cell toxicity screening. Cells were cultured in DMEM (Gibco, USA) supplemented with 10% fetal bovine serum (FBS; Gibco, USA), 100 U mL^−1^ penicillin, and 100 μg mL^−1^ streptomycin at 37 °C in a 5% CO₂ humidified incubator. Cells were seeded into 96-well plates at a density of 1 × 10^4^ cells well^−1^ and incubated for 24 h.

The extracts were added at concentrations of 31.25–500 μg mL^−1^ and incubated for 72 h. After treatment, cells were washed with PBS and incubated with 0.5 mg mL^−1^ MTT solution (Sigma-Aldrich, USA) for 3 h. Formazan crystals were dissolved in 100 μL of DMSO, and absorbance was measured at 570 and 650 nm using a microplate reader (BioTek, USA). Doxorubicin was used as a positive control to confirm assay performance and sensitivity. All experiments were conducted in triplicate, and results are expressed as mean ± standard deviation (SD).

### 2.9 Sensory evaluation

This study was assessed and approved by the Chiang Mai University Research Ethics Committee (CMUREC No. 68/043) on February 11, 2025. All participants acknowledged an informed consent declaration to participate in the study. The Declaration of Helsinki, the International Conference on Harmonization in Good Clinical Practice (ICH-GCP), and The Belmont Report were followed. Sensory evaluation procedures were carried out following the methodology described by Xu et al. (2025).

The fermented beverage produced was classified as a functional drink, characterized by a distinct color and characteristic flavor derived from CC, along with mild acidity. For sensory evaluation, four formulations were selected: (1) unfermented CC, (2) CC fermented with *L.* *plantarum* TISTR 2070 (LP2070), (3) CC fermented with *S. boulardii* CNCM I-745 (SB745), and (4) CC fermented with LP2070 and SB745 (co-culture). All samples were stored at 4 °C and served chilled (4–6 °C) to the panelists.

The sensory panel consisted of 50 panelists, aged 20 to 50 years, all experienced in food sensory evaluation. The participants employed a hedonic scale ranging from 1 (lowest) to 9 (highest) to evaluate appearance, color, smell, texture, mouthfeel, acidity, bitterness, sweetness, and overall acceptability. Sensory data were expressed as mean ± standard deviation (SD), and statistical significance among formulations was assessed using the methods described in Section 2.10 (*p* < 0.05).

### 2.10 Analysis of statistics

All assays were performed in biological triplicate (*n* = 3). Data are reported as mean ± standard deviation (SD). One-way analysis of variance (ANOVA) followed by Tukey–Kramer post hoc test was used to determine significant differences (*p* < 0.05) using SPSS v17.0 (IBM, USA). Assumptions of normality and homogeneity of variance were assessed using the Shapiro–Wilk and Levene's tests, respectively. Response surface modelling and lack-of-fit analyses were conducted using Design-Expert v13.0 (Stat-Ease Inc., USA).

## 3 Results and discussion

### 3.1 Single-culture fermentations

#### 3.1.1 Viability of single-culture fermentations

**Fig. 1:**
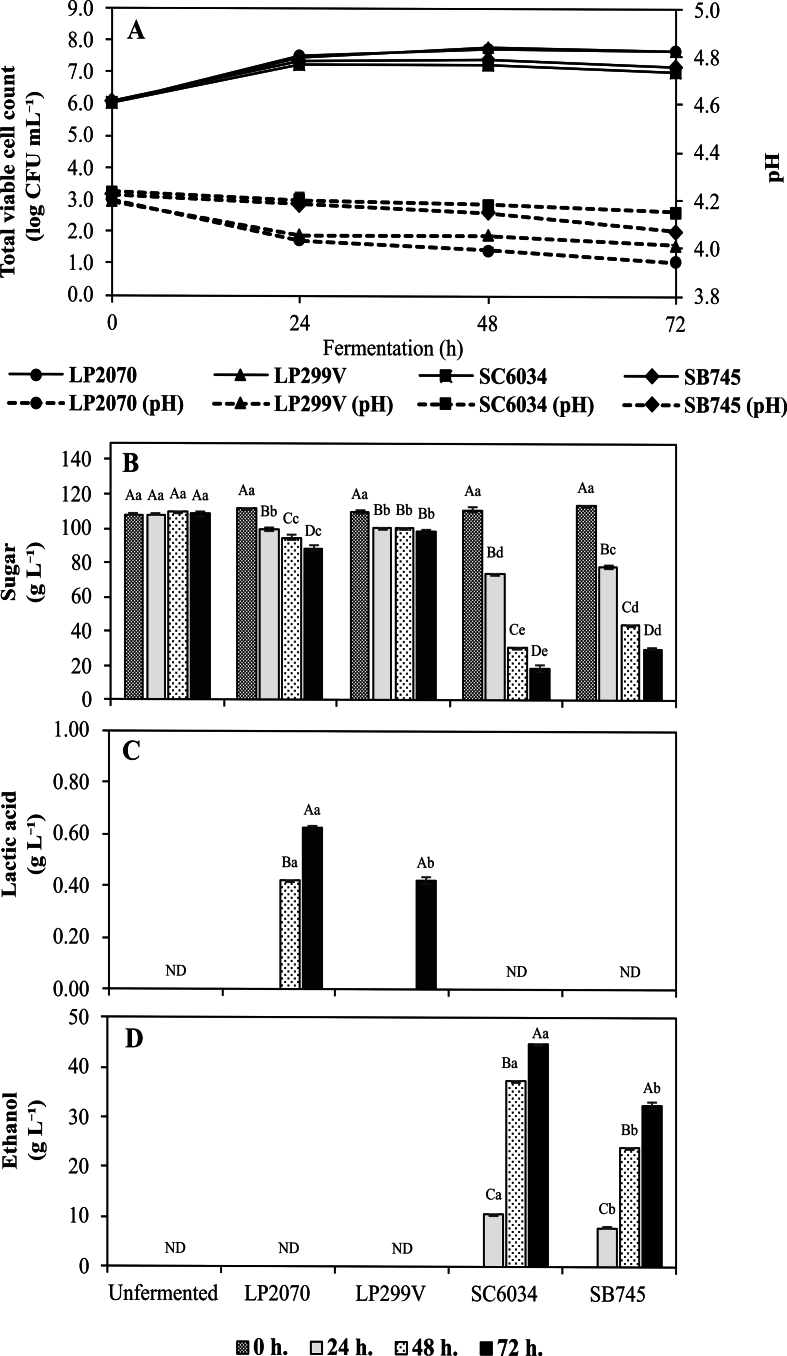
Viable cell counts and pH (A), sugar concentrations (B), lactic acid (C), and ethanol (D) during single-culture fermentation of coffee cherry pulp extract. Values are expressed as mean ± SD of *n* = 3 independent experiments. Strain abbreviations: LP2070 = *L. plantarum* TISTR 2070; LP299V = *L. plantarum* 299v; SC6034 = *S. cerevisiae* TISTR 6034; SB745 = *S. boulardii* CNCM I-745. Different uppercase letters indicate significant differences within the same starter across fermentation times (*p* < 0.05). Different lowercase letters indicate significant differences among starters within the same fermentation time (*p* < 0.05).

At 24 h fermentation, *L. plantarum* TISTR 2070 (LP2070) and *L*. *plantarum* 299v (LP299V) exhibited the highest viable cell counts, measuring 7.50 ± 0.03 log CFU mL^−1^, followed by *S. boulardii* CNCM I-745 (SB745) at 7.38 ± 0.05 log CFU mL^−1^ and *S. cerevisiae* TISTR 6034 (SC6034) at 7.22 ± 0.07 log CFU mL^−1^ **(**Fig. 1A**)**. A reduction in viable cell numbers was observed at 48 h. By 72 h, viable cell populations slightly decreased further. However, most strains largely maintained high counts, with SC6034 declining significantly to 6.86 ± 0.09 log CFU mL^−1^, whereas the other strains-maintained counts above 7 log CFU mL^−1^.

Although the viable cell counts of *L*. *plantarum* and *Saccharomyces* spp. showed a slight decline after 48–72 h of fermentation, they remained above 6 log CFU mL^−1^, a threshold considered sufficient to confer health benefits by the probiotic approach (Mojikon et al., 2022). *L. plantarum* TISTR 2070 exhibited stability under stress conditions, such as high polyphenols and organic acids concentrations. Several studies have reported the ability of LAB to grow and survive well in fruit-based fermentation systems rich in phenolics and organic acids, including sea grape juice co- fermented with coffee cherry pulp (Moukamnerd et al., 2025) and other acidic fruit juices (Tuerhong et al., 2024).

In addition, SB745 has a remarkable growth ability. As reported by Souza et al. (2024), in a fermentation system with water kefir for 9 days, *S. boulardii* produced a cell count as high as 9 log CFU mL^−1^, which is higher than that of conventional *S. cerevisiae* strains. Therefore, the observed reduction in viable cell count in both LAB and yeast does not indicate deterioration but rather represents a natural transition into the stationary phase, which is still compatible with the functional criteria required for health-oriented fermented beverages.

#### 3.1.2 pH, sugar, lactic acid, and ethanol of single-culture fermentations

Monitoring the changes in pH, sugar content, lactic acid, and ethanol is critical for evaluating the metabolic activities during fermentation. High-performance liquid chromatography (HPLC) analysis revealed a significant decrease in total sugar concentration (sum of sucrose, glucose, and fructose) as fermentation progressed **(**Fig. 1B**)**. The unfermented CC extract was naturally acidic (pH 4.20 ± 0.04) and was not adjusted prior to inoculation. This native acidity favors the growth of acid-tolerant LAB and yeast while reducing the risk of spoilage and pathogenic contamination. After 24 h, LP2070 produced lactic acid at concentrations up to 0.62 ± 0.02 g L^−1^ **(**Fig. 1C**)**, leading to a marked pH drops to 3.94 ± 0.06 **(**Fig. 1A**)**. The pH continued to decline gradually from 24 to 72 h, albeit at a slower rate, indicating continuous lactic acid production. In yeast fermentations, SC6034 produced the highest ethanol concentration (44.67 ± 0.01 g L^−1^) after 72 h **(**Fig. 1D**)**, indicating efficient sugar to ethanol conversion.

The reduction in sugar levels, coupled with lactic acid accumulation by LP2070, reflects efficient carbohydrate utilization and subsequent acidification, creating unfavorable conditions for spoilage microorganisms and thereby enhancing product stability (de Melo Pereira et al., 2020). Simultaneously, the ethanol yield of SC6034 aligns with previously reported values (∼31.0 g L^−1^) from fresh coffee cherry fermentation using selected commercial yeast strains (Einfalt et al., 2020). SB745 produced significantly lower ethanol concentrations, which is consistent with earlier findings and likely attributable to its metabolic preference for biomass formation over ethanol production (Souza et al., 2024).

#### 3.1.3 Total phenolic and antioxidant activity of single-culture fermentations

**Fig. 2:**
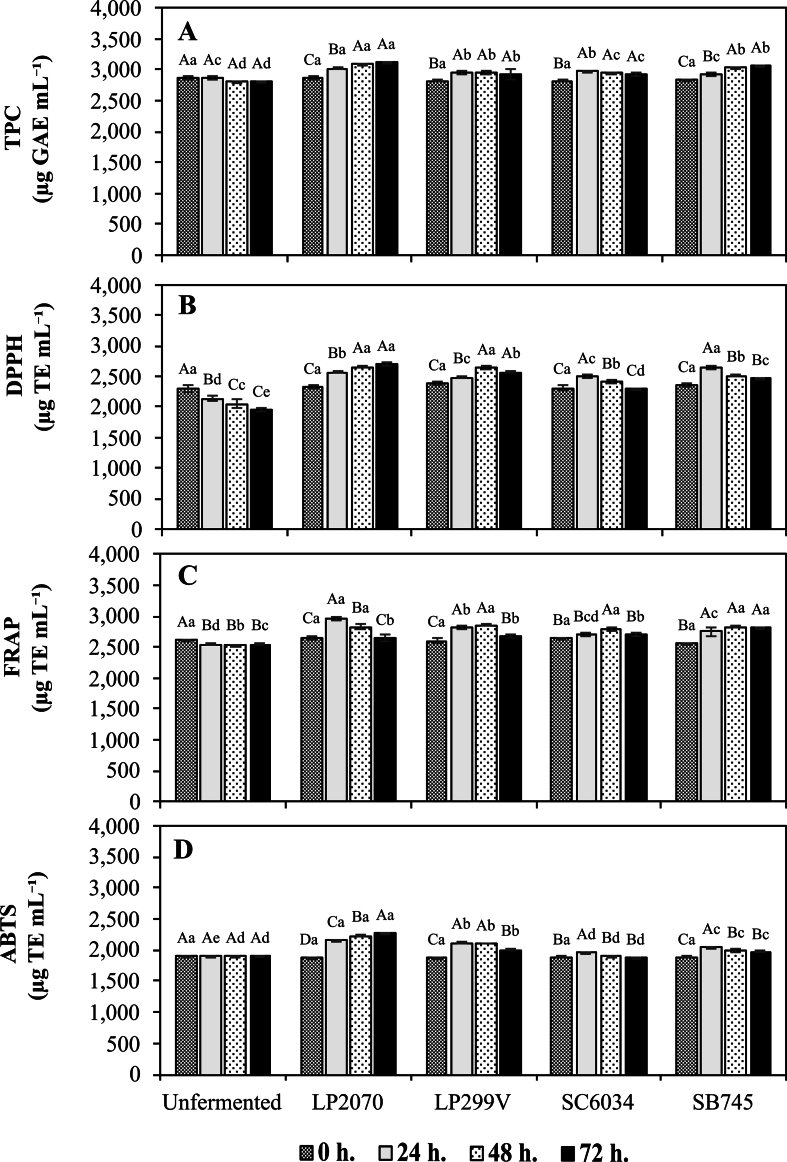
The total phenolic content (A), antioxidant activity; DPPH (B), FRAP (C), and ABTS (D) during single-culture fermentation of coffee cherry pulp extract. Values are expressed as mean ± SD of *n* = 3 independent experiments. Strain abbreviations: LP2070 = *L. plantarum* TISTR 2070; LP299V = *L. plantarum* 299v; SC6034 = *S. cerevisiae* TISTR 6034; SB745 = *S. boulardii* CNCM I-745. Different uppercase letters indicate significant differences within the same starter across fermentation times (*p* < 0.05). Different lowercase letters indicate significant differences among starters within the same fermentation time (*p* < 0.05).

To identify optimal strains for co-culture fermentation, total phenolic content (TPC) and antioxidant activity were used as primary selection criteria. Among LAB strains, LP2070 exhibited the highest TPC **(**Fig. 2A**)**, and was significantly higher (*p* < 0.05) than the other screened LAB strain (*L. plantarum* 299v) at the 24 h mark. Specifically, LP2070 produce TPC significantly increased (*p* < 0.05) to 3023.61 ± 24.55 μg GAE mL^−1^. Correspondingly, antioxidant activities assessed via DPPH, FRAP, and ABTS assays showed notable increases to 2569.15 ± 34.13 μg TE mL^−1^, 2974.49 ± 28.86 μg TE mL^−1^, and 2155.23 ± 10.07 μg TE mL^−1^, respectively **(**Fig. 2B-D**).**

For yeast strains, SB745 demonstrated a continuous increase in total phenolic content (TPC) throughout the fermentation period (24–72 h), reaching 3075 ± 28.0 μg GAE mL^−1^ at 72 h and maintaining statistically superior levels (*p* < 0.05) compared to SC6034 thereafter. In contrast, SC6034 exhibited higher TPC than SB745 only at 24 h, after which its phenolic content declined progressively. Moreover, SB745 achieved the highest antioxidant activity at 24 h, with DPPH, FRAP, and ABTS values of 2654.26 ± 30.09, 2753.40 ± 62.53, and 2047.39 ± 2.31 μg TE mL^−1^, respectively (Fig. 2B–D), consistently outperforming SC6034 across all antioxidant indices.

The increase in TPC and antioxidant activity after fermentation with LP2070 reflects the potential of LAB to transform complex compounds into more bioactive forms, especially the hydrolysis of chlorogenic acid to caffeic acid and quinic acid through esterase enzymes produced by LAB (Myo et al., 2021). This result is consistent with the report by Moukamnerd et al. (2025), which demonstrated that LP2070 markedly enhanced TPC and antioxidant activity in a co-fermentation system involving sea grapes and coffee cherry pulp.

Conversely, SB745 demonstrated the ability to hydrolyze glycosidic bonds in phenolic compounds through the β-glucosidase enzyme, which aligns with the findings of Chan et al. (2021), who reported increased bioactive compounds following the fermentation of coffee-based substrates. Similarly, Souza et al. (2024) showed that fermentation with *S. boulardii* enhanced total phenolic content (TPC) and DPPH radical scavenging activity in plant-based beverages, likely due to the enzymatic biotransformation of this yeast strain.

Given their superior and statistically confirmed performance among the four screened strains in total phenolic content and antioxidant enhancement, LP2070 and SB745 were selected for co-culture fermentation. Therefore, LP299V and SC6034 were not included in the RSM-based co-culture optimization. The selection of these specific strains provides a foundation for more precise control over the release of phenolic compounds and enables the design of highly efficient co-culture fermentation systems aimed at enhancing bioactive compounds, addressing the inherent variability often associated with complex microbial consortia in natural fermentations (Patil et al., 2022).

### 3.2 Co-culture fermentations

#### 3.2.1 RSM-based optimization of co-culture conditions to maximize phenolic and antioxidant yields

To systematically identify optimal fermentation parameters for enhancing bioactive compound production, a statistical optimization approach using Response Surface Methodology (RSM) with a Box–Behnken Design (BBD) was employed (Therdtatha et al., 2023). This model was designed to evaluate the impact of four critical variables: LP2070 inoculum level (A), SB745 inoculum level (B), fermentation time (C), and incubation temperature (D) on total phenolic content (TPC) and antioxidant activity, measured via DPPH, FRAP, and ABTS assays.

The multiple regression analysis confirmed the significance of the quadratic models for all response variables, as evidenced by a statistically significant Model F-value (*p* < 0.05) and coefficients of determination (R^2^ and adjusted R^2^) ranging from 0.919 to 0.943 and 0.838 to 0.887, respectively (Table 2), indicating strong model fit. Furthermore, the lack-of-fit was non-significant, confirming the model's reliability (Gomes-Lobo et al., 2025). The high Adequate Precision values (ranging from 10.815 to 13.227), which significantly exceed the typical threshold of 4.0, also confirmed that the models possess sufficient signal-to-noise ratio for reliable prediction within the experimental design space. The fundamental assumptions for ANOVA, including normality and homogeneity of variance, were also satisfied, as confirmed by diagnostic plots **(Fig. S1)**, further supporting the statistical validity of the models.

**Table 2:** Quadratic equations derived from BBD for the predictions.

Response variables	Quadratic models	Model F-value	Model *p*-value	*R*^*2*^	*R*^*2*^_*adj.*_	Adequacy Precision
TPC (μg GAE mL^−1^)	3230.91 + 28.39 A + 50.58B + 149.01C + 52.81D – 73.71A^2^–111.26B^2^–191.64C^2^–76.41D^2^–61.03AB + 26.22 AC + 39.93 CE - 1.74 BCE + 42.10BD + 20.83CD	11.35	0.0001	0.919	0.838	10.895
DPPH (μg TE mL^−1^)	3123.03 + 25.49 A + 72.40B + 183.58C + 3.69D -165.61A^2^–315.76B^2^–264.23C^2^–170.59D^2^ + 5.54AB + 29.92 AC + 9.97 CE + 18.84 BCE - 57.62BD -21.05CD	12.70	0.0001	0.927	0.854	12.397
FRAP (μg TE mL^−1^)	3183.61 + 13.62 A + 60.37B + 160.73C – 18.14D -173.42A^2^–176.43B^2^–243.58C^2^–74.81D^2^ + 45.92AB + 38.32 AC + 16.16 CE + 28.06 BCE + 51.02BD + 2.55CD	16.63	0.0001	0.943	0.886	13.227
ABTS (μg TE mL^−1^)	2228.37 + 9.79 A + 24.87B + 189.21C – 3.91D + 7.06A^2^–35.64B^2^–154.63C^2^–20.07D^2^–11.44AB -5.27 AC - 11.85 CE + 10.37 BCE + 60.46BD - 1.75CD	16.64	0.0001	0.943	0.887	11.865

A, B, C, and D represent the independent variables of the experimental design, where A is the inoculum level of *L.plantarum* LP2070 (log CFU mL^−1^), B is the inoculum level of *S. boulardii* SB745 (log CFU mL^−1^), C is the fermentation time (h), and D is the incubation temperature (°C).

Under optimized conditions (LP2070 at 7.30 log CFU mL^−1^, SB745 at 2.52 log CFU mL^−1^, 30 °C, 26 h), the co-culture system achieved significantly elevated TPC (3354.17 ± 39.58 μg GAE mL^−1^) and antioxidant activities: DPPH (3091.76 ± 15.79 μg TE mL^−1^), FRAP (3198.98 ± 14.43 μg TE mL^−1^), and ABTS (2259.80 ± 6.93 μg TE mL^−1^), surpassing both unfermented and predicted values (Table 3).

**Table 3:** Experimental validation of the suggested optimal conditions from BBD.

Treatments	Experimental conditions				Experimental results			
	LP2070 (log CFU mL^−1^)	SB745 (log CFU mL^−1^)	Time (h)	Temperature (°C)	TPC (μg GAE mL^−1^)	DPPH (μg TE mL^−1^)	FRAP (μg TE mL^−1^)	ABTS (μg TE mL^−1^)
Unfermented CC	–	–	–	–	2808.33 ± 14.17	2457.89 ± 50.58	2637.76 ± 33.65	1867.65 ± 10.59
Predicted optimal conditions	7.12	2.38	26.23	30.55	3256.40	3138.78	3201.12	2249.48
Experimental validation	7.30 ± 0.02	2.52 ± 0.05	26.00	30.00	3354.17 ± 39.58	3091.76 ± 15.79	3198.98 ± 14.43	2259.80 ± 6.93
Predicted error (%)	–	–	–	–	+3.00%	−1.50%	−0.07%	+0.46%

Predicted optimal conditions from BBD by mathematic model (Set maximum value for TPC and antioxidant activities responses)

In addition, ethanol content was maintained at a low level (1.40% *v*/v ABV; 11.03 ± 0.09 g L^−1^), classifying the beverage as a low-alcohol product suitable for general consumption and compatible with acceptable sensory characteristics.

These results confirm that RSM-guided co-culture optimization is an effective strategy to maximize the functional attributes of fermented coffee cherry pulp.

#### 3.2.2 Interaction effects and threshold dynamics in co-culture fermentation systems

**Fig. 3:**
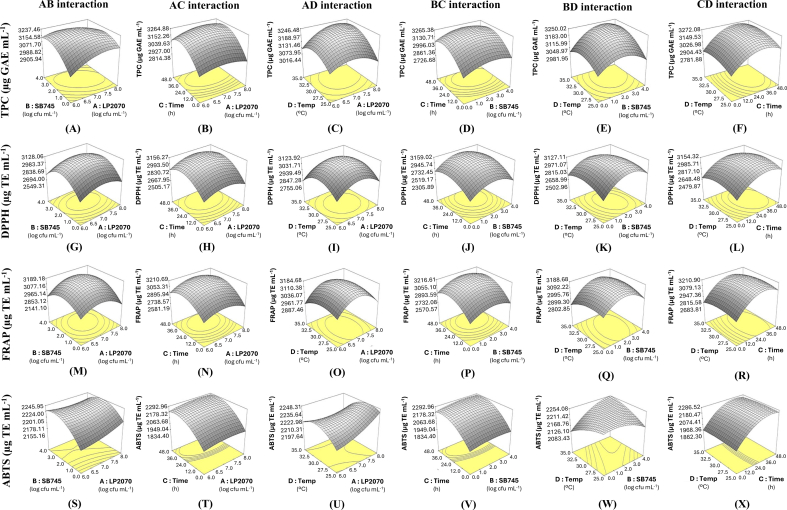
Three-dimensional plots illustrating the interactive effects of two independent factors on total phenolic content (TPC) (A–F), DPPH (G–L), FRAP (M–R), and ABTS (S–X) in coffee cherry pulp extract fermented by co-culture. The plot shows the interactions between LP2070 and SB745 (AB), LP2070 and time (AC), LP2070 and temperature (AD), SB745 and time (BC), SB745 and temperature (BD), and time and temperature (CD).

Beyond individual effects, the interaction among fermentation parameters played a critical role in modulating phenolic transformation and antioxidant capacity. While SB745 (B) demonstrated a strong individual effect on responses, LP2070 (A) showed significant interactions with both fermentation time (C) and temperature (D), particularly in AC and AD interactions **(**Fig. 3B–C**)**, suggesting a modulatory role of lactic acid in enhancing phenolic compound release (Zhang et al., 2025).

Incubation temperature (D) also exhibited synergistic effects with SB745, notably enhancing ABTS and FRAP activities (BD interactions in Fig. 3W and Q), highlighting the temperature sensitivity of *S. boulardii* for enzyme activation and metabolite production (Wang et al., 2022). These findings align with previous reports that identified 30 °C as an optimal temperature for *S. boulardii* bioactivity.

Quadratic response analysis revealed non-linear behavior across all variables, with excessive inoculum levels, fermentation durations, or elevated temperatures leading to a decline in bioactive yields. This phenomenon represents a biological threshold or saturation effect, where nutrient depletion, acidification, or metabolite accumulation negatively impacts microbial viability and enzymatic efficiency (Yuan et al., 2025). For instance, certain combinations of high inoculum levels led to a measurable drop in TPC and antioxidant values, suggesting stress-induced suppression of compound biosynthesis.

These results underscore the importance of fine-tuning microbial ratios and process parameters to maintain metabolic balance, avoid overstimulation, and achieve optimal co-culture synergy. Such balance is essential to maximize bioactive compound release, preserve microbial health, and minimize off-flavor formation in functional fermented beverages.

### 3.3 In vitro inhibition of α-amylase and α-glucosidase by co-culture fermented beverage

The co-culture fermentation exhibited the highest inhibitory activity against α-amylase and α-glucosidase, with *IC*_*50*_ values of 4.67 ± 0.08 mg mL^−1^ and 3.13 ± 0.05 mg mL^−1^, respectively **(**Table 4**)**. These results clearly demonstrate an enhancement of bioactivity attributed to the co-fermentation process. Although single-culture fermentations also exhibited enzyme-inhibitory potential, their activities were significantly lower (*p* < 0.05) compared to the co-culture system.

**Table 4:** *IC*_*50*_ values (mg mL^−1^) of fermented and unfermented coffee cherry pulp (CC) extracts against α-amylase and α-glucosidase.

Experiment	*IC*_*50*_ (mg mL^−1^)	
	α-amylase	α-glucosidase
Unfermented	8.05 ± 0.44 ^A^	5.26 ± 0.05 ^A^
LP2070	6.19 ± 0.09 ^C^	3.65 ± 0.07 ^C^
SB745	6.46 ± 0.12 ^B^	4.00 ± 0.02 ^B^
Co-culture	4.67 ± 0.07 ^D^	3.13 ± 0.05 ^D^
Positive control	2.21 ± 0.00 ^E^	2.30 ± 0.01 ^E^

Values are expressed as mean ± SD of *n* = 3 independent experiments. Acarbose (5 mg mL^−1^) was used as the positive control. Different letters within columns indicate significant differences (*p* < 0.05) based on Tukey's multiple comparison test.

Compared to previous studies using hot-water extraction coupled with high-pressure steaming, the fermented extracts from this study presented significantly lower *IC*_*50*_ values, indicating superior enzyme inhibitory efficiency (Alyahya et al., 2021; Patil et al., 2022). In contrast, semi-purified, commercial-grade extracts derived from whole coffee cherry pulp have reported even lower *IC*_*50*_ values of 1.74 and 2.42 mg mL^−1^ for α-amylase and α-glucosidase, respectively (Nemzer et al., 2021). These methods, however, require complex ethanol–water purification steps, resulting in higher production costs and limited scalability.

The co-culture fermentation strategy proposed here offers a natural, food-compatible and cost-efficient approach for producing bioactive beverages, positioning it as a promising and scalable alternative for the development of functional products with α-glucosidase and α-amylase inhibitory potential.

### 3.4 LC-QTOF-MS based metabolite profiling of functional beverage

Qualitative LC-QTOF-MS analysis (Table 5) revealed that both single- and co-culture fermentations with *L.* *plantarum* 2070 and *S. boulardii* 745 generated a diverse array of bioactive metabolites, putatively identified (MSI Level 2) via high matching scores (70%) from accurate-mass and MS/MS spectral matching. These metabolites are mechanistically associated with enhanced antioxidant activities and inhibition of carbohydrate-hydrolyzing enzymes.

**Table 5:** Determination of bioactive compound by LC-QTOF-MS.

No.	Proposed Compounds	Molecular Formula	Unfermented		LP2070		SB745		Co-culture	
			Mass	Matching score (%)	Mass	Matching score (%)	Mass	Matching score (%)	Mass	Matching score (%)
	**Organic acids**									
1	Citric acid	C_6_ H_8_ O_7_	ND	–	ND	–	ND	–	191.0172	92.63
2	Pantothenic acid	C_9_ H_17_ N O_5_	ND	–	218.1032	86.92	218.1034	86.07	218.1031	86.36
3	Phenyllactic acid	C_9_ H_10_ O_3_	ND	–	167.0706	82.43	167.0706	87.08	167.0706	86.03
4	Quinic acid	C_7_ H_12_ O_6_	191.0556	83.10	191.0557	79.34	191.0558	85.28	191.0558	85.96
5	*p*-Hydroxyphenylacetic acid	C_8_ H_8_ O_3_	151.0397	76.27	151.0397	84.48	151.0397	84.96	151.0398	85.63
6	Sebacic acid	C_10_ H_18_ O_4_	201.1130	86.29	201.1128	83.97	201.113	82.50	201.1130	85.13
7	D-Glucuronic acid	C_6_ H_10_ O_7_	ND	–	ND	–	193.0354	86.96	193.0360	84.94
8	Phloionolic acid	C_18_ H_36_ O_5_	331.2484	82.46	331.2488	85.01	331.249	98.78	331.2489	84.66
9	Fukiic acid	C_11_ H_12_ O_8_	271.0455	92.89	271.0460	85.62	271.0452	83.93	271.0454	84.36
10	Phenylmalonic acid	C_9_ H_8_ O_4_	ND	–	ND	–	ND	–	181.0501	81.24
11	Indolelactic acid	C_11_ H_11_ N O_3_	ND	–	204.0662	78.18	ND	–	204.0661	81.51
	**Amino acids and peptides**									
12	Glutamyl-Gamma-glutamate	C_10_ H_16_ N_2_ O_7_	ND	–	ND	–	275.1104	96.41	275.1110	99.44
13	L-Valine	C_5_ H_11_ N O_2_	118.0864	87.31	118.0864	99.38	ND	–	118.0864	87.22
14	L-isoleucyl-L-proline	C_11_ H_20_ N_2_ O_3_	ND	–	229.155	86.05	229.1552	78.68	229.1551	85.77
15	Alanine	C_3_ H_7_ N O_2_	ND	–	ND	–	655.1924	70.26	655.1922	84.07
16	Vanilloylglycine	C_10_ H_11_ N O_5_	ND	–	ND	–	ND	–	226.0713	82.74
	**Polyphenols and Phenolic acids**									
17	3,4,5-Trihydroxybenzaldehyde	C_7_ H_6_ O_4_	153.0193	99.98	153.0192	99.76	153.0192	99.91	153.0193	99.91
18	1,5-Dicaffeoylquinic acid	C_25_ H_24_ O_12_	515.1196	78.93	ND	–	515.1197	91.86	515.1195	99.39
19	Methyl chlorogenate	C_17_ H_20_ O_9_	ND	–	369.1190	79.44	369.1186	81.66	367.1036	99.24
20	Caffeic acid	C_9_ H_8_ O_4_	180.0420	83.83	180.0420	80.69	180.0419	85.80	180.0414	82.49
21	2-Ethoxy-5-(1-propenyl)phenol	C_11_ H_14_ O_2_	179.1071	71.84	179.1071	85.69	179.1071	85.48	179.1072	84.80
22	Salicylic acid β-D-glucoside	C_13_ H_16_ O_8_	299.0770	72.01	299.0771	85.01	299.0770	77.22	299.0771	84.06
23	2,4-Dihydroxybenzoic acid	C_7_ H_6_ O_4_	ND	–	155.0341	98.94	ND	–	137.0238	83.77
24	m-Trigallic acid	C_21_ H_14_ O_13_	ND	–	ND	–	ND	–	473.0370	75.86
	**Alkaloids**									
25	Tabernine B	C_16_ H_17_ N_3_	ND	–	ND	–	ND	–	250.1348	99.36
26	8-Benzyloxycaffeine	C_15_ H_16_ N_4_ O_3_	ND	–	323.1109	93.06	ND	–	323.1112	87.09
	**Coumarin**									
27	6-Hydroxycoumarin	C_9_ H_6_ O_3_	163.0395	83.35	163.0395	98.22	163.0394	98.37	163.0394	98.66
	**Other**									
28	Triptophenolide	C_20_ H_24_ O_3_	311.1656	92.87	ND	–	ND	–	309.1737	94.73

Acquisition was performed in positive ionization mode; however, some metabolites were annotated as negative-ion species ([M–H]^−^) based on library/database matching.

Polarity, adduct form, and matching score (≥ 70%) are indicated in the table. Compound identification was based on accurate mass (Δ ≤ 5 ppm), isotopic distribution, retention time, and MS/MS spectral similarity. Higher matching scores reflect greater confidence in compound identification. ND indicates not detected.

To facilitate mechanistic interpretation, metabolites were grouped functionally into (i) polyphenols and phenolic acids, (ii) organic acids and amino-acid derivatives, and (iii) peptides and related compounds. This functional classification, which prioritizes occurrence patterns (e.g., metabolites uniquely generated in co-culture), allows us to explore how microbial interactions reprogram metabolic networks through microbial interactions, leading to functionally enhanced metabolite profiles.

(i) Polyphenols and Phenolic Acids.

1,5-dicaffeoylquinic acid (1,5-DCQA; [M – H]^−^, *m/z* 515.1195) exhibited the highest matching score (99.39%) in the co-culture extract while being absent in LP2070. This pattern strongly indicates metabolic complementarity and cross-feeding between LP2070 and SB745, where enzymatic activities from both microorganisms synergistically facilitate biosynthesis or prevent degradation of 1,5-DCQA. MS/MS fragments at *m/z* 353.0874 and 191.0555 are consistent with the loss of one caffeoyl moiety and formation of quinic acid ion **(Fig. S4)**. Literature supports 1,5-DCQA as a potent α-glucosidase inhibitor (Lee et al., 2022), providing a biochemical explanation for the lower IC₅₀ values observed in co-culture extracts (Table 4).

Methyl chlorogenate (MChl; [M – H]^−^, *m/z* 367.1036) appeared in all fermented samples but was absent in the unfermented control. Its formation likely involves co-expressed microbial esterases and methyltransferases, with MS/MS fragments at *m/z* 191.0557 and 179.0345 consistent with the formation of quinic and caffeic acid ions **(Fig. S8)**. Furthermore, 3,4,5-trihydroxybenzaldehyde ([M – H]^−^, *m/z* 153.0192) was across all samples **(Fig. S6)**, indicating its stability as an intrinsic phenolic compound in the CC. Together, these results demonstrate that *co*-culture fermentation enhances both the diversity and overall abundance of bioactive polyphenols, linking directly to observed antioxidants and enzyme-inhibitory activities.

(ii) Organic Acids and Amino-Acid Derivatives.

Co-culture fermentation uniquely produced citric acid ([M – H]^−^, *m/z* 191.0172, matching score 92.63%) and m-trigallic acid ([M – H]^−^, *m/z* 473.0370, matching score 75.86%), both of which were absent in single-culture fermentations. The accumulation of citric acid suggests metabolic shifts in carbon utilization, through the modulation of the TCA cycle activity or enhanced citrate production export by the co-culture strains. Alternatively, its origin could involve the synergistic catabolism of plant-derived citrate from the coffee cherry pulp, which is preferentially released or accumulated by the microbial interaction. This unique production is functionally significant: Citric acid is a strong α-glucosidase inhibitor (Noh et al., 2020), noting its potential role in influencing enzyme structure or binding. Meanwhile, m-trigallic acid, a polymer of gallic acid likely formed through cooperative microbial polymerization, provides potent dual antioxidant and diabetic enzyme inhibitory activity (Aleixandre et al., 2022). The synthesis of m-trigallic acid, a complex phenolic polymer, is hypothesized to be mediated by the cooperative action of microbial laccase or peroxidase enzymes, which are potentially co-expressed by the LP2070 and SB745 strains under co-culture conditions.

Additional metabolites such as indolelactic acid (ILA) and phenyllactic acid (PLA) reflect active tryptophan and phenylalanine catabolism, likely mediated by microbial stress responses (Maione et al., 2025). Pantothenic acid (vitamin B₅) detected in all fermented samples suggests de novo biosynthesis, though confirmation would require genomic or enzymatic evidence from the specific strains (LP2070 and SB745). D-glucuronic acid, present only in SB745 and co-culture samples, may derive from yeast metabolism or plant-matrix degradation; isotope-tracing experiments would clarify its source**.** These findings highlight mechanistic links between microbial interactions and the production of high-value organic acids contributing to both antioxidant and enzyme-inhibitory activities.

(iii) Amino Acids and Peptides.

The proteolytic activity of LP2070, coupled with enzymatic cooperation with SB745, released amino acids and small peptides, including L-Isoleucyl-L-Proline and Glutamyl-γ-glutamate ([M + H]+, *m/z* 275.1110, matching score 99.44%). These compounds were more abundant in co-culture extracts. MS/MS analysis further supported the dipeptide structure, with a major fragment observed at *m/z* 257.09 (consistent with the loss of a water molecule) and other characteristic fragments supporting peptide backbone cleavage. Peptides derived from branched-chain amino acids are implicated in radical-scavenging and enzyme-inhibitory mechanisms (Canon et al., 2022). Their enrichment indicates enhanced proteolytic and peptidogenic metabolism achieved through microbial cooperation.

Comprehensive profiling and critical safety screening utilizing LC-QTOF-MS confirmed the absence of pesticide residues (including organophosphates, pyrethroids, and carbamates) in the fermented extract. Crucially, no signal corresponding to methanol or other potentially harmful short-chain alcohols was detected above the Limit of Detection (LoD), supporting the overall safety profile of the developed product.

(iv) Correlation between Metabolites and Biological Activities.

Pearson correlation analysis (*n* = 12) revealed strong positive correlations between total phenolic content and antioxidant activities (DPPH, FRAP, ABTS; *r* = 0.88–0.98, *p* < 0.01). Conversely, α-amylase/α-glucosidase IC₅₀ values showed significant negative correlations with TPC (*r* = −0.966 and −0.954, respectively; *p* < 0.01). These findings provide mechanistic support for the link between metabolite enrichment and observed bioactivities, suggesting polyphenol–organic acid synergy and contributions from bioactive peptides **(Table S1)**.

Co-culture fermentation reprograms the metabolic network of coffee cherry pulp through interconnected microbial interactions, bioconversion, and de novo synthesis. Cross-feeding, enzymatic cooperation, and stress-induced metabolism promote the formation of high-value compounds (e.g., 1,5-DCQA, MChl, citric acid, m-trigallic acid, pantothenic acid, and bioactive peptides), whose functional synergy enhances antioxidant activity and α-glucosidase inhibition.

This mechanism-driven framework, supported by MS/MS evidence and correlation analysis, provides a comprehensive explanation for the superior functional performance of co-culture extracts, highlighting their potential as next-generation functional beverage ingredients.

### 3.5 Cytotoxicity test of functional beverage

All tested samples (unfermented, single culture (LP2070 and SB745), and co-culture fermented CC extracts) exhibited *IC*_*50*_ values greater than 500 μg mL^−1^, indicating no cytotoxic effect within the tested concentration range (31.25–500 μg mL^−1^). Doxorubicin served as a positive control (*IC*_*50*_ = 20.01 ± 0.49 μM). The absence of cytotoxicity at 500 μg mL^−1^ indicates a conservative safety margin for systemic exposure, as this concentration exceeds levels expected to arise from realistic beverage consumption. The lack of cytotoxicity further suggests the absence of harmful compounds, such as toxic alcohols, at levels of concern. However, further in vivo studies and clinical trials are warranted to confirm the absence of renal toxicity and ensure safety for human consumption.

The coffee cherry pulp used in this study was sourced from Thep Sadet, Chiang Mai Province, Thailand and extracted using the same hot-water method and solid-to-solvent ratio previously described by Chomphoosee et al. (2025). In their study, fermentation using water kefir grains also resulted in no cytotoxicity against normal human colon epithelial cells at concentrations up to 10,000 μg mL^−1^. Although different cell lines were used (renal epithelial vs. colon epithelial), both are non-tumorigenic and commonly employed for evaluating the safety of natural products. The consistent absence of cytotoxicity across studies, despite variations in fermentation microbiota and cell models, reinforces the biocompatibility and safety of CC-based fermented beverages and supports their potential application as functional food ingredients.

### 3.6 Sensory evaluation of functional beverage

A sensory evaluation was conducted to assess consumer acceptance of functional beverages produced from coffee cherry pulp (CC). Four formulations were tested: (1) unfermented CC, (2) CC fermented with *L.* *plantarum* TISTR 2070 (LP2070), (3) CC fermented with *S. boulardii* CNCM I-745 (SB745), and (4) CC co-culture fermented with LP2070 and SB745. The detailed attribute means, standard deviations, and statistical differences are provided in **Table S2**.

**Fig. 4:**
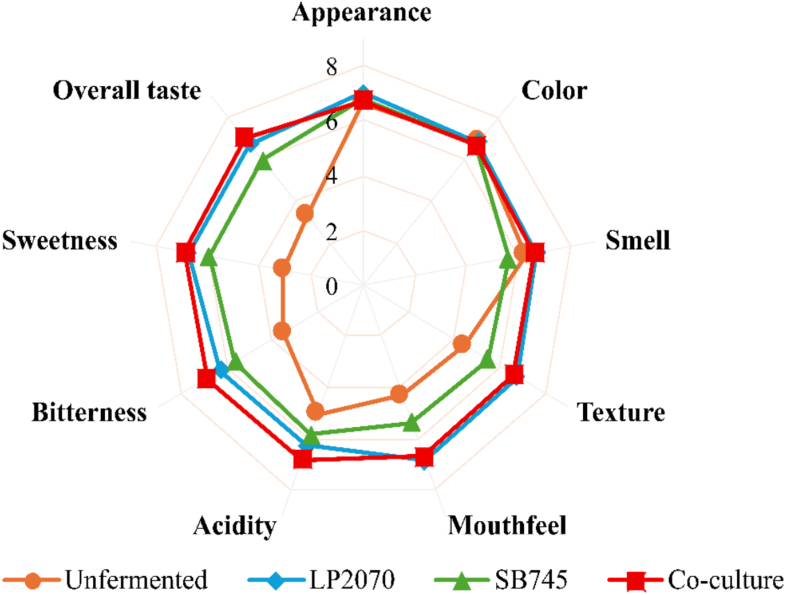
Sensory evaluation of the fermented coffee cherry pulp beverages.

As shown in Fig. 4, The co-culture fermented sample achieved the highest overall acceptability score (7.54 ± 1.16), which was significantly higher (*p* < 0.05) than LP2070 (6.78 ± 1.27), SB745 (6.26 ± 1.14), and the unfermented control (3.58 ± 1.34). Significant improvements in liking scores (*p* < 0.05) were observed across all fermented samples.

In particular, the co-culture formulation showed significantly higher scores for sweetness (6.90 ± 1.30), bitterness (6.88 ± 1.20), mouthfeel (6.70 ± 1.25), and texture (6.62 ± 1.26) compared to the unfermented control, indicating the most pronounced enhancement in overall flavor perception.

The enhanced acceptance achieved through fermentation involved a trade-off in visual attributes. While the unfermented sample received the highest color liking score (6.82 ± 1.42), the co-culture formulation was rated significantly lower (6.68 ± 1.27), indicating that improved flavor and overall liking came at the expense of visual appeal. This observation is supported by colorimetric analysis **(Table S3)**, which showed a significant reduction in lightness (L) accompanied by increased redness (a) and yellowness (b*) in the co-culture sample, consistent with fermentation-induced pigment modification.

These objective measurements confirm that co-fermentation drastically altered the natural pigment composition, resulting in a darker, more saturated hue that was less preferred by the sensory panel, despite the product's superior flavor profile.

Metabolic activities of the fermenting microorganisms contributed to enhanced sensory perception. *L. plantarum* plays a pivotal role in converting sugars into organic acids and functional metabolites that enhance perceived sweetness and reduce bitterness, thus improving overall flavor balance. In addition, *S. boulardii* CNCM I-745 significantly contributes to aroma development through the biosynthesis of volatile compounds, enhancing overall flavor complexity (Wang et al., 2022).

### 3.7 Multivariate analysis

**Fig. 5:**
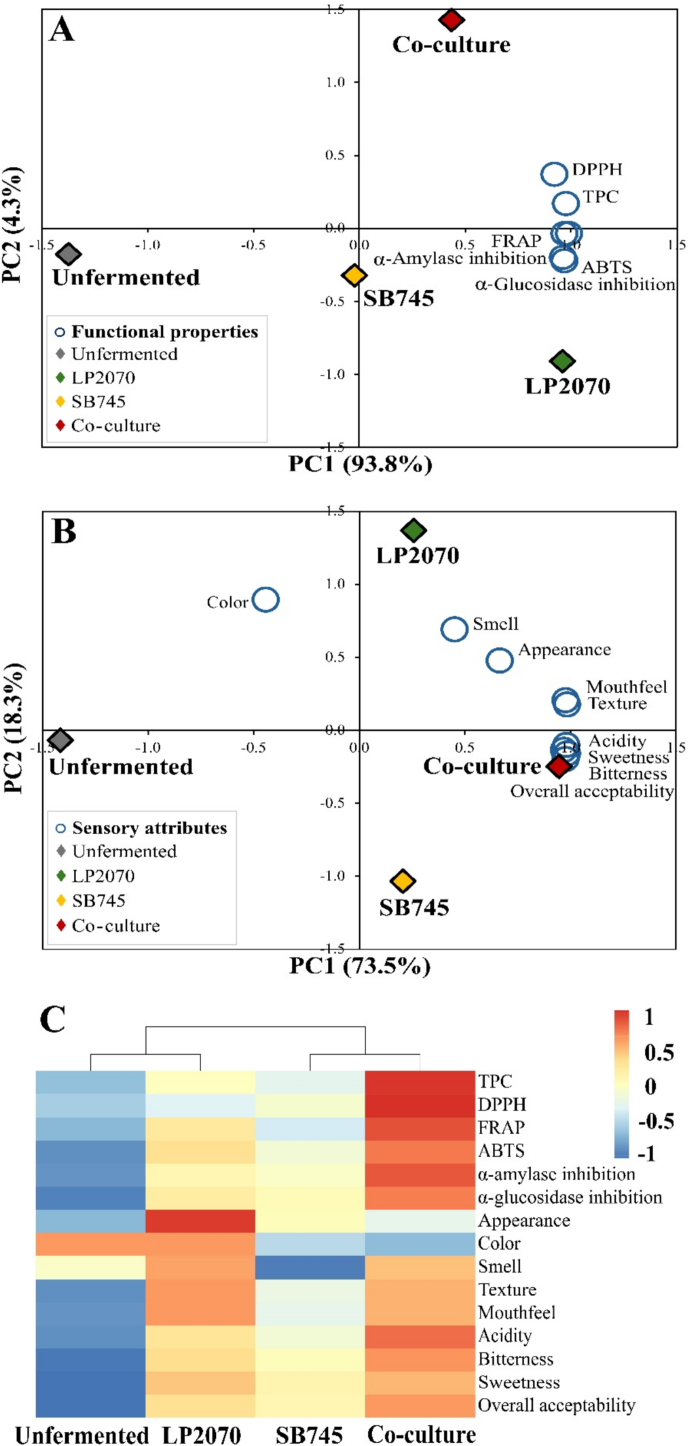
Principal component analysis (PCA) biplot; Functional properties (A), Sensory attributes (B), and hierarchical clustering heatmap (C) of fermented coffee cherry pulp extracts.

PCA analysis of functional properties (TPC, DPPH, FRAP, ABTS, α-amylase, and α-glucosidase inhibition) showed that PC1 explained 93.8% of the variance and PC2 added 4.3% **(**Fig. 5A**)**. The co-culture clearly separated from the other groups on the positive side of the PC1 axis and had a close relationship with the antioxidant and enzyme inhibition assays, indicating higher functional properties. Furthermore, the unfermented and single-fermentation samples were positioned on the negative end of the PC1 axis.

For sensory evaluation of the functional beverage by PCA showed that PC1 accounted for 73.5% of the variance, while PC2 explained an additional 18.3%, together representing 91.8% of the cumulative variance **(**Fig. 5B**)**. Co-culture fermentation was positively associated with sensory attributes related to sweetness, bitterness, and overall acceptability. These findings suggest that the synergistic interaction between LP2070 and SB745 effectively improved consumer taste perception and satisfaction. These patterns are consistent with the univariate sensory results **(**Fig. 4**)**, confirming the enhanced acceptability of co-culture fermentation.

In addition, PCA analysis corresponded with the heat map graph **(**Fig. 5C**)**, demonstrating that co-culture fermentation positively influenced nutritional benefits and enhanced sensory balance. This enhancement may lead to increased consumer acceptability of its application as a functional beverage.

## 4 Conclusion

The optimized co-culture fermentation of coffee cherry pulp (CC) with *L. plantarum* TISTR 2070 (LP2070) and *S. boulardii* CNCM I-745 (SB745) effectively promoted the release of phenolic compounds, improved both antioxidant capacity, high in vitro α-amylase and α-glucosidase inhibitory activity achieved high sensory acceptability. LC-QTOF-MS profiling uncovered a diverse range of health-promoting metabolites. Cytotoxicity testing further confirmed the non-toxic nature of the fermented CC extract in mammalian cells, reinforcing the safety of the final product.

This bioprocess demonstrates practical scalability potential, as it employs widely available starter cultures and conventional fermentation infrastructure. Nonetheless, further optimization will be required during scale-up to address process-related challenges, including heat and mass transfer efficiency and the maintenance of consistent microbial performance. In addition, the favorable sensory acceptability observed at laboratory scale should be validated through shelf-life studies to ensure quality stability during storage.

Overall, these findings support co-culture fermentation as a sustainable strategy for valorizing agricultural residues into functional ingredients. Future studies should focus on in vivo bioavailability and efficacy, along with techno-economic and shelf-life evaluations, to support industrial translation and integration into circular bioeconomy systems.

## CRediT authorship contribution statement

**Supanut Pothimoi:** Writing – original draft, Validation, Methodology, Investigation, Data curation. **Phisit Seesuriyachan:** Writing – review & editing. **Thanongsak Chaiyaso:** Writing – review & editing, Conceptualization. **Chayatip Insomphun:** Writing – review & editing. **Kamon Yakul:** Writing – review & editing. **Yuthana Phimolsiripol:** Writing – review & editing. **Churairat Moukamnerd:** Writing – review & editing, Supervision, Project administration, Conceptualization.

## Ethical statement

This study was reviewed and approved by the Chiang Mai University Research Ethics Committee (CMUREC No. 68/043) on February 11, 2025. All procedures involving human participants were conducted in accordance with institutional guidelines and the Declaration of Helsinki. Written informed consent was obtained from all participants prior to their involvement in the study.

## Declaration of competing interest

The authors declare that they have no known competing financial interests or personal relationships that could have appeared to influence the work reported in this paper.

## Data Availability

The data supporting this study's findings are available from the corresponding author upon reasonable request.

## Appendix A Supplementary data

Supplementary material 1 Image 1

Supplementary material 2 Image 2
