# Supplementary material for: Enhanced sensory, antioxidant, and non-toxic anti-diabetes beverage development via co-culture fermentation of Lactiplantibacillus plantarum and Saccharomyces boulardii in coffee cherry pulp extracts
Source: Food Chem X. 2026 Jan 12;33:103524. doi: 10.1016/j.fochx.2026.103524 (PMC12853062; doi:10.1016/j.fochx.2026.103524)
Supplement: Supplementary file 1 — Supplementary material 1 [file mmc1.docx]

Enhanced Sensory, Antioxidant, and Non-Toxic Anti-Diabetes Beverage Development via Co-Culture Fermentation of *Lactiplantibacillus plantarum* and *Saccharomyces boulardii*
in Coffee Cherry Pulp Extracts

Supanut Pothimoi ^b^, Phisit Seesuriyachan ^a,c^, Thanongsak Chaiyaso ^a^, Chayatip Insomphun ^a^,

Kamon Yakul ^a^, Yuthana Phimolsiripol ^a^, Churairat Moukamnerd ^a,c,^*

*^a^ School of Agro-Industry, Faculty of Agro-Industry, Chiang Mai University, Chiang Mai, 50100, Thailand*

*^b^ Interdisciplinary Program in Biotechnology, Multidisciplinary and Interdisciplinary School,*

*Chiang Mai University, Chiang Mai, 50200, Thailand*

*^c^ Advanced Technology and Innovation Management for Creative Economy Research Group (AIMCE), Department of Industrial Engineering, Faculty of Engineering, Chiang Mai University, Chiang Mai, 50200, Thailand*

* Corresponding author: Churairat Moukamnerd (E-mail: churairat.m@cmu.ac.th)

**
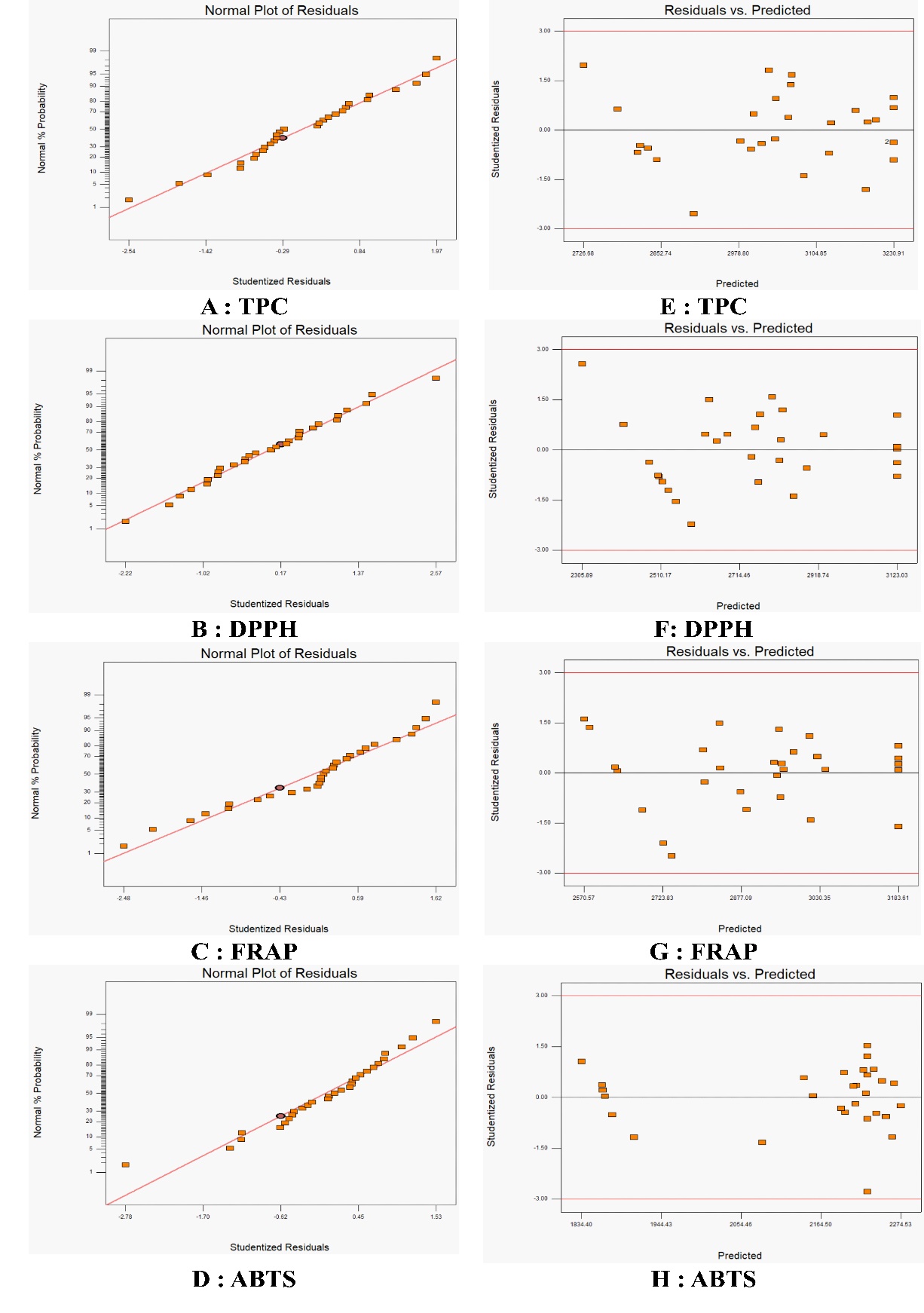
**

**Figure S1.** Residual diagnostic plots for the fitted quadratic models of the four responses. (A–D) Normal probability plots of studentized residuals showing that the residuals follow an approximately normal distribution. (E–H) Residuals vs. predicted plots showing random scatter with no funnel-shaped pattern, confirming homoscedasticity. All residuals fall within the ±3 studentized limits, indicating no strong outliers.


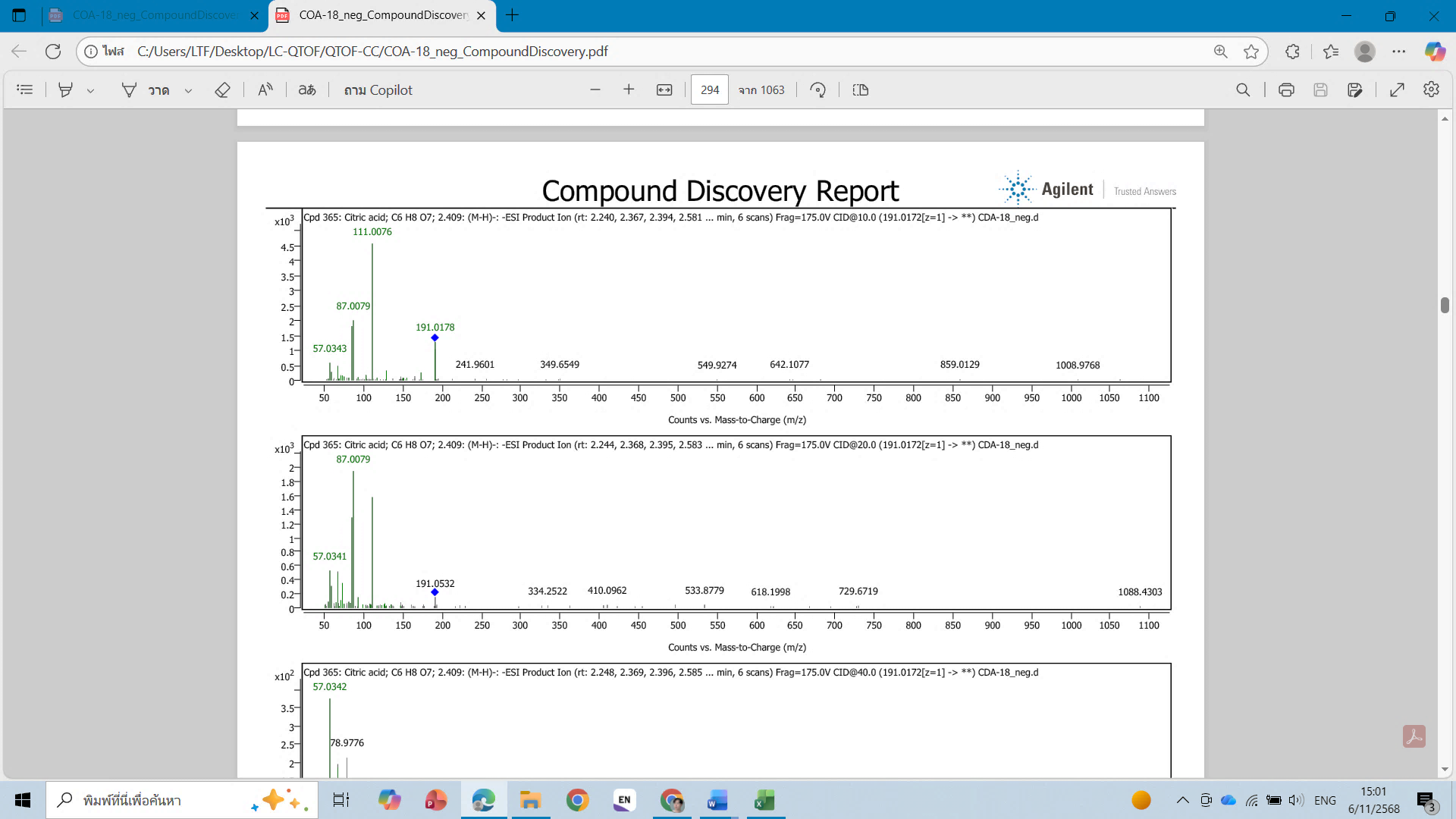


**Figure S2.** Negative ion MS/MS spectrum of citric acid ([M – H] ⁻, m/z 191.0172) acquired at 20 eV collision energy. Major diagnostic fragments were observed at m/z 87.0079 and 57.0341, corresponding to the characteristic sequential decarboxylation and dehydration steps of the citric acid backbone, in agreement with reported fragmentation pathways in metabolic databases. Library match score 92.63%.


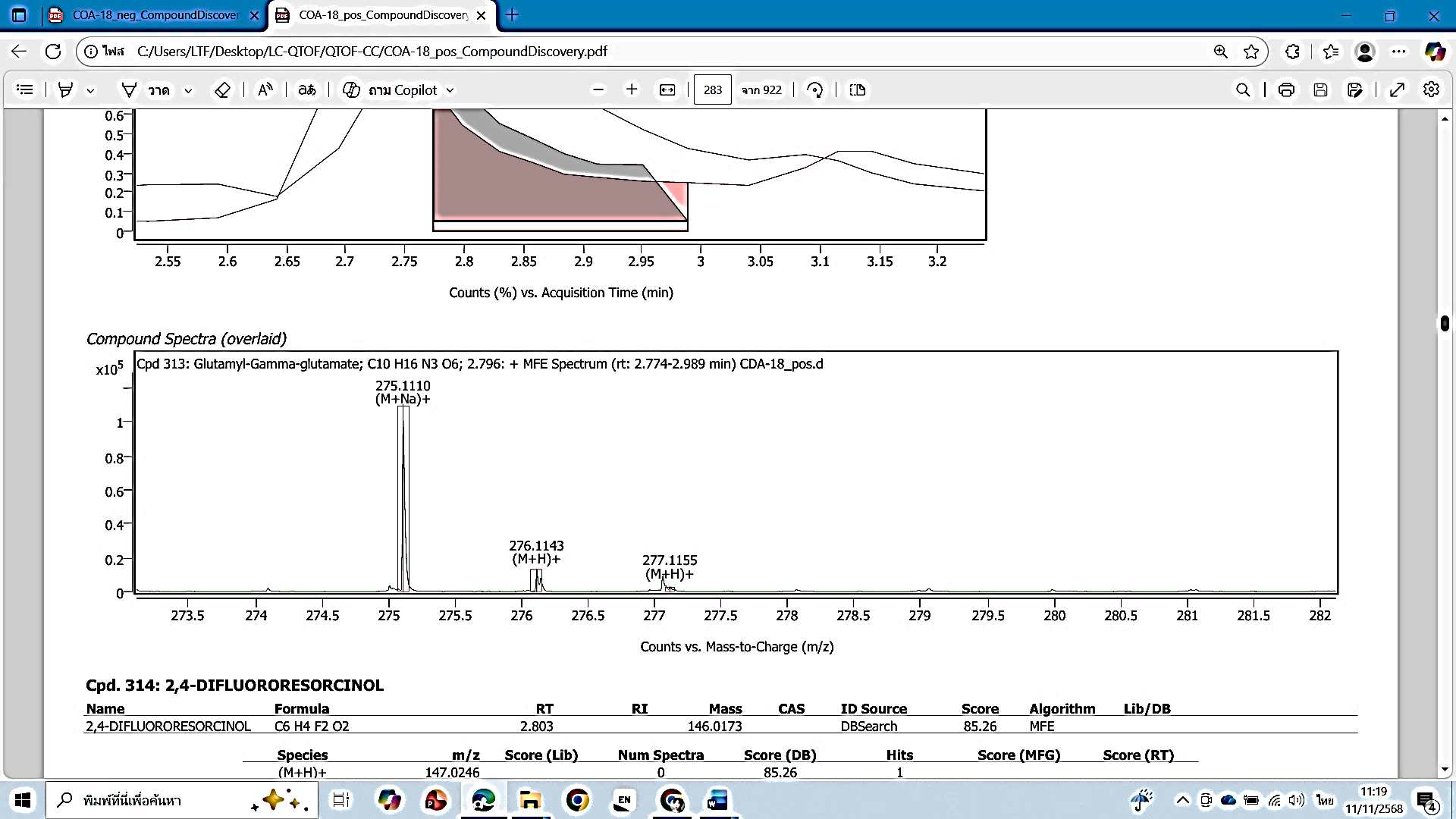


**Figure S3.** Positive ion MS/MS spectrum of Glutamyl-Gamma-glutamate. The precursor ion, [M + H]^+^, was observed at m/z 275.1110 (accurate mass from the table is 275.1110/275.1104 Da) and the compound eluted at a retention time (RT) of approximately 2.796 minutes. A major diagnostic fragment was observed at m/z 257.09 (corresponding to the loss of a water molecule), with other fragments supporting the cleavage of the peptide backbone. Library match score 99.44%.


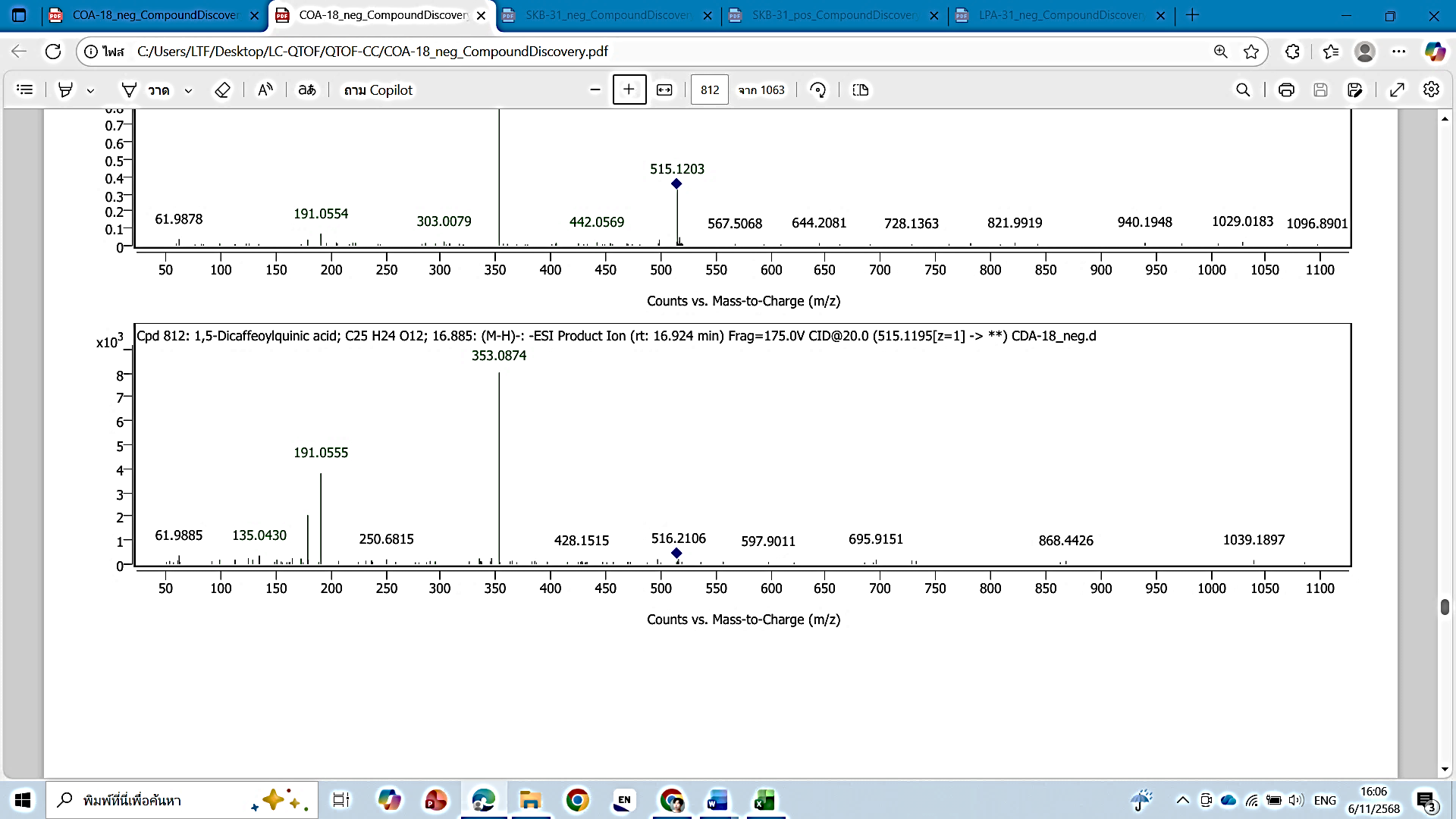


**Figure S4.** Negative ion MS/MS spectrum of 1,5-dicaffeoylquinic acid ([M – H] ⁻, m/z 515.1195) acquired at 20 eV collision energy. Major diagnostic fragments were observed at m/z 353.0874 and 191.0555, corresponding to the neutral loss of one caffeoyl moiety and the formation of the quinic acid ion, respectively, consistent with the characteristic fragmentation pattern of dicaffeoylquinic acid isomers reported in metabolite databases. Library match score 95.88%.


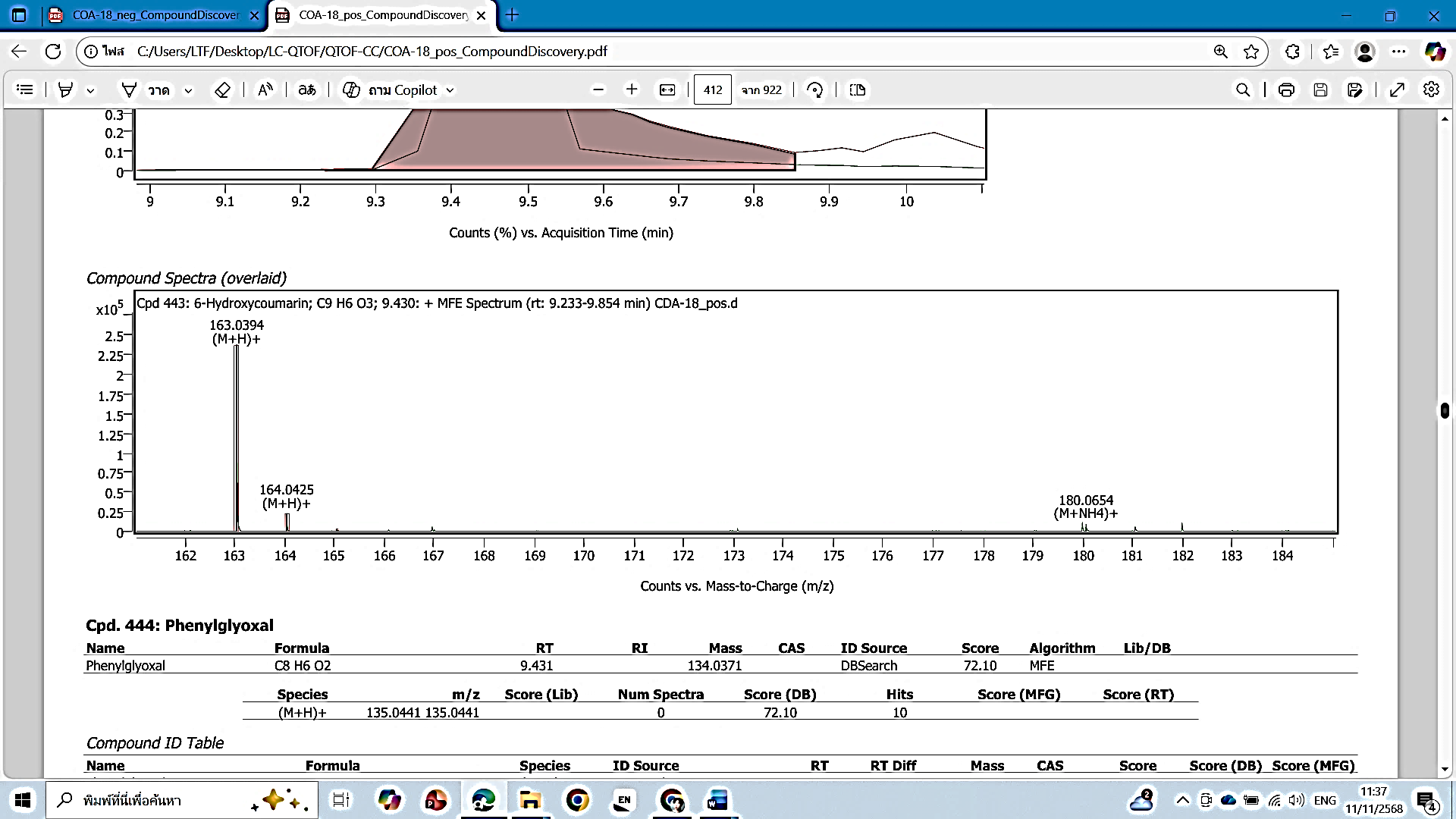


**Figure S5.** Positive ion MS/MS spectrum of 6-Hydroxycoumarin. The precursor ion, [M + H]+, was observed at m/z 163.0394 (accurate mass is 163.0390 Da) and the compound eluted at a retention time (RT) of approximately 9.430 minutes. Major diagnostic fragments were observed at m/z 135.0441 and m/z 107.0492, corresponding to the sequential neutral loss of two molecules of carbon, which is characteristic of the fragmentation pattern of coumarin derivatives. Library match score: 98.66%.


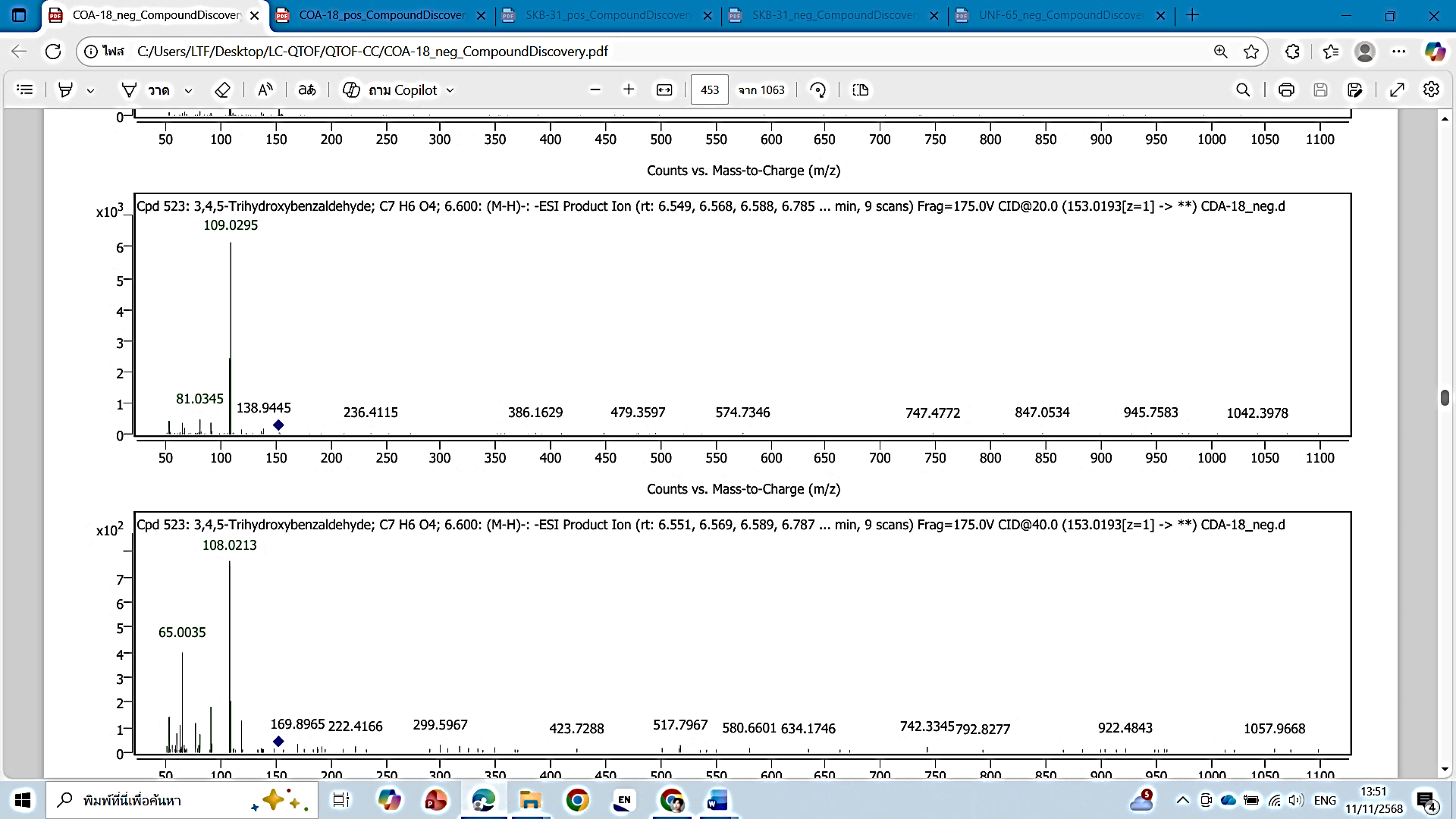


**Figure S6.** Negative ion MS/MS spectrum of 3,4,5-Trihydroxybenzaldehyde. The precursor ion, [M – H]⁻, was observed at m/z 153.0193. The compound eluted at a retention time (RT) of approximately 6.600 minutes. The spectrum was acquired using a fragmentation voltage (Frag) of 175.0 V and a collision energy (CID) of 20 eV. A major diagnostic fragment was observed at m/z 109.0298, corresponding to the neutral loss of carbon dioxide, which is characteristic of the fragmentation pattern of Trihydroxybenzaldehyde derivatives. Library match score: 99.98%.


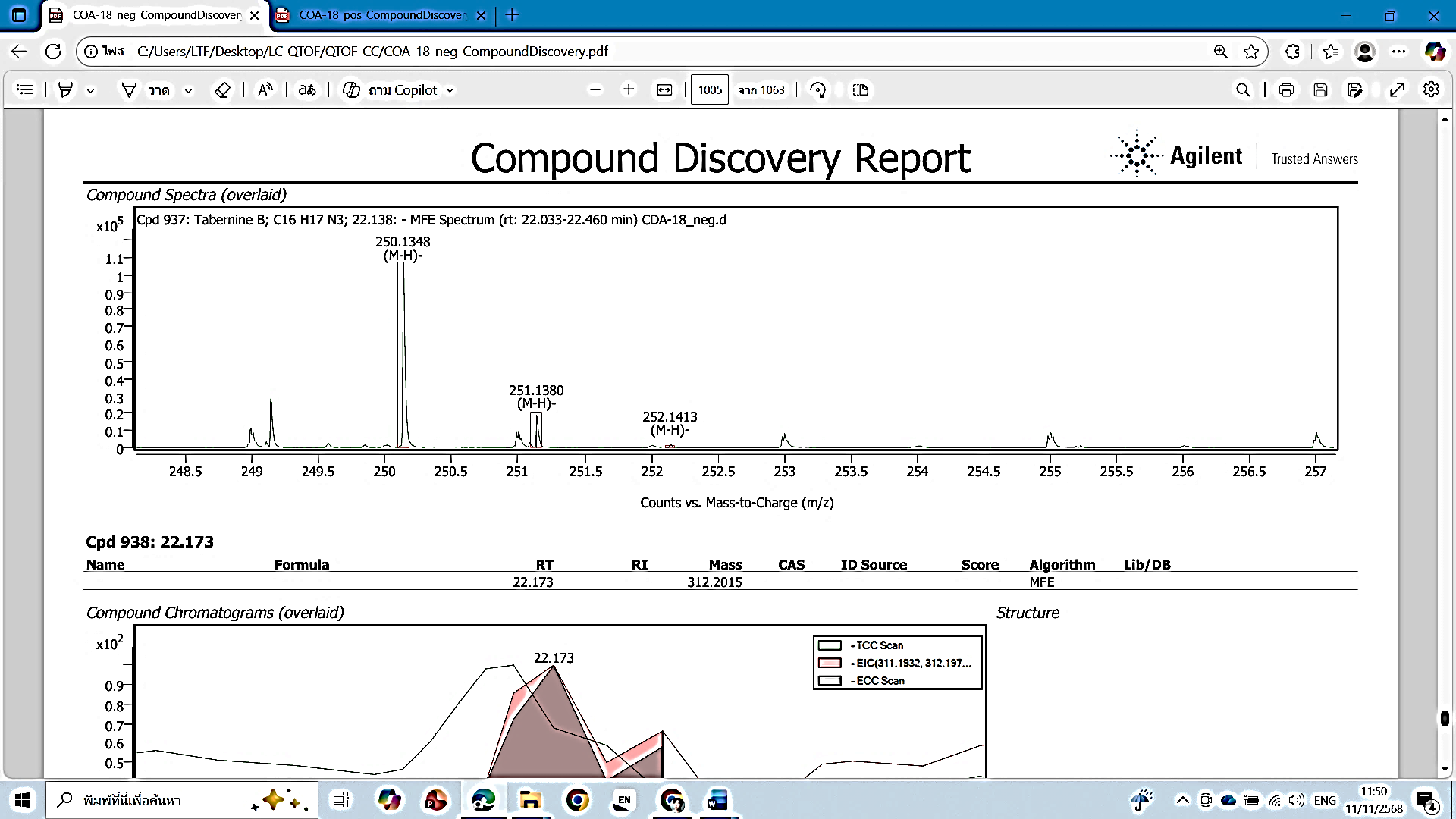


**Figure S7.** Negative ion MS/MS spectrum of Tabernine B. The precursor ion, [M – H] ⁻, was observed at m/z 250.1345 (accurate mass is 250.1345 Da) and the compound eluted at a retention time (RT) of approximately 22.138 minutes. The spectrum was acquired using the MFE Spectrum acquisition method. Major diagnostic fragment was observed at m/z 235.1110, corresponding to the neutral loss of a methyl radical, which is characteristic of the fragmentation pattern of indole alkaloids, such as tabernanthine derivatives. Library match score: 99.36%.


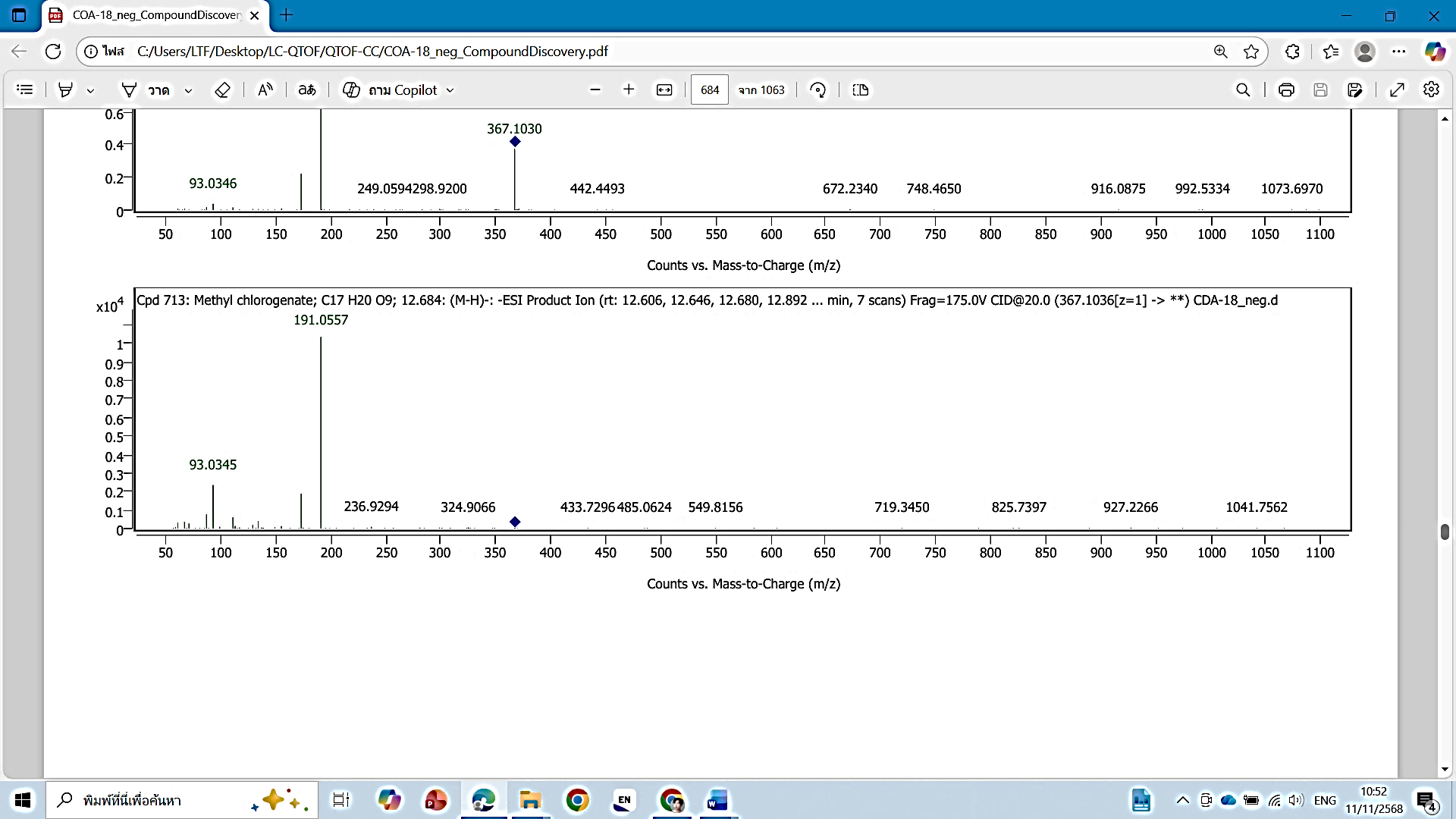


**Figure S8.** Negative ion MS/MS spectrum of Methyl chlorogenate ([M – H]⁻, m/z 367.1036) acquired at 20 eV collision energy. Major diagnostic fragments were observed at m/z 191.0557 and m/z 179.0345, corresponding to the formation of the quinic acid ion and the caffeic acid ion, respectively, consistent with the characteristic fragmentation pattern of chlorogenic acid derivatives. Library match score: 99.24%.


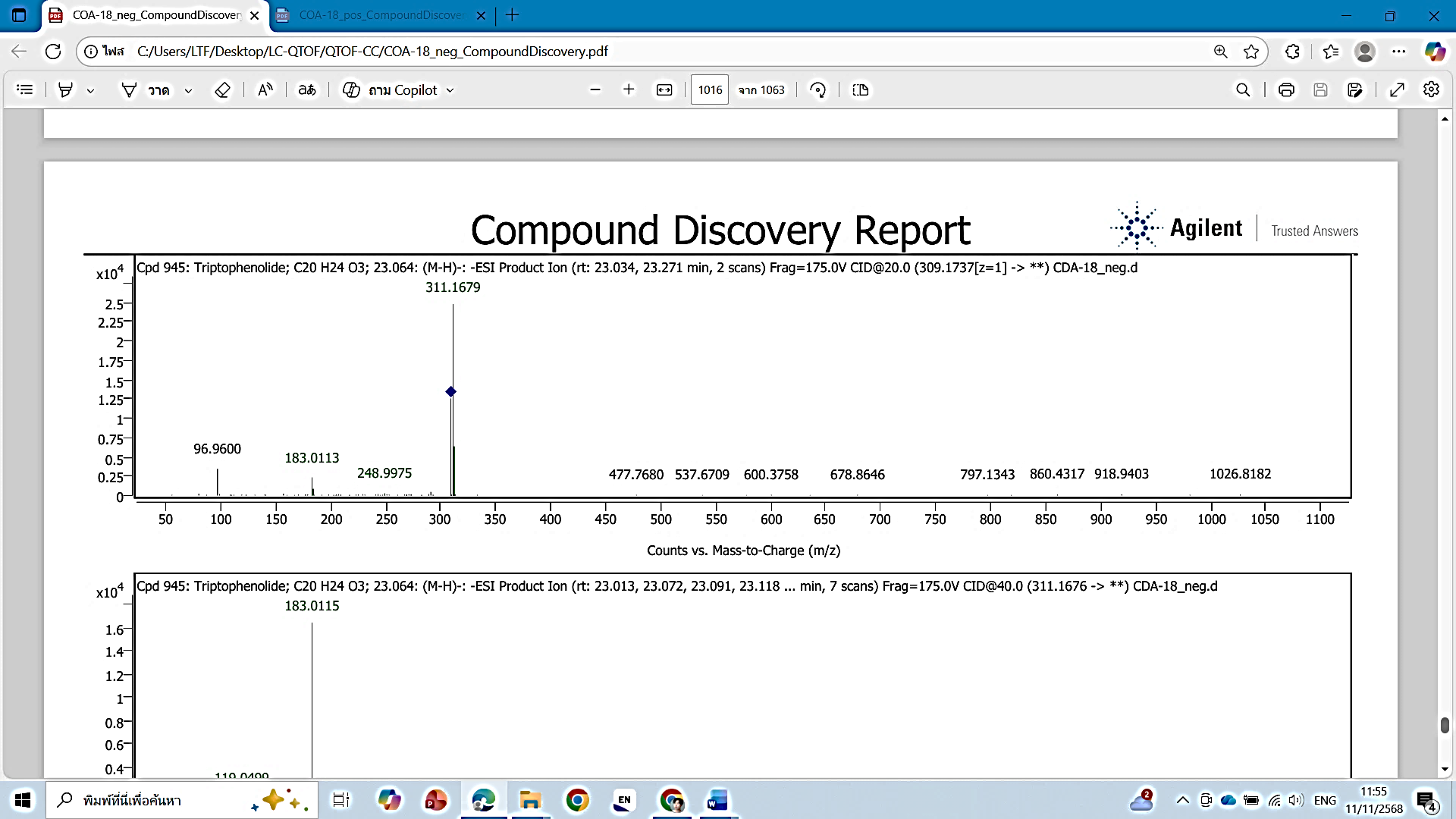


**Figure S9.** Negative ion MS/MS spectrum of Triptophenolide. The precursor ion, [M – H]⁻, was observed at m/z 309.1737. The compound eluted at a retention time (RT) of approximately 23.064 minutes. The spectrum was acquired using a fragmentation voltage (Frag) of 175.0 V and a collision energy (CID) of 20 eV. Major diagnostic fragments were observed at m/z 273.1539, corresponding to the neutral loss of two molecules of water, which is consistent with the characteristic fragmentation pattern of diterpenoids. Library match score: 94.73%.

**Table S1.** Pearson correlation coefficients (r) among TPC, antioxidant indices, and enzyme inhibition (n = 12).

| Variables | TPC | DPPH | FRAP | ABTS | IC₅₀  α-amylase | | IC₅₀  α-glucosidase | |
| --- | --- | --- | --- | --- | --- | --- | --- | --- |
| TPC | 1 | 0.964** | 0.962** | 0.964** | | -0.966** | | -0.954** |
| DPPH | 0.964** | 1 | 0.981** | 0.899** | | -0.942** | | -0.891** |
| FRAP | 0.962** | 0.981** | 1 | 0.880** | | -0.964** | | -0.894** |
| ABTS | 0.964** | 0.899** | 0.880** | 1 | | -0.932** | | -0.980** |
| IC₅₀ α-amylase | -0.966** | -0.942** | -0.964** | -0.932** | | 1 | | 0.954** |
| IC₅₀ α-glucosidase | -0.954** | -0.891** | -0.894** | -0.980** | | 0.954** | | 1 |
| Correlation is significant at the 0.01 level (two-tailed).  Data points (n = 12) were derived from four treatments (unfermented, LP2070, SB745, and  co-culture), each with three biological replicates. | | | | | | | | |

| Sensory Attribute | Unfermented | LP2070 | SB745 | Co-culture |
| --- | --- | --- | --- | --- |
| Appearance | 6.64 ± 1.51 ^d^ | 7.00 ± 1.31 ^a^ | 6.80 ± 1.16 ^b^ | 6.74 ± 1.21 ^c^ |
| Color | 6.82 ± 1.42 ^a^ | 6.82 ± 1.34 ^a^ | 6.70 ± 1.39 ^b^ | 6.68 ± 1.27 ^b^ |
| Smell | 6.26 ± 1.37 ^c^ | 6.70 ± 1.33 ^a^ | 5.62 ± 1.32 ^d^ | 6.62 ± 1.29 ^b^ |
| Texture | 4.44 ± 1.40 ^d^ | 6.76 ± 1.20 ^a^ | 5.48 ± 1.37 ^c^ | 6.62 ± 1.26 ^b^ |
| Mouthfeel | 4.32 ± 1.36 ^d^ | 6.86 ± 1.28 ^a^ | 5.36 ± 1.27 ^c^ | 6.70 ± 1.25 ^b^ |
| Acidity | 5.04 ± 1.64 ^d^ | 6.28 ± 1.65 ^b^ | 5.86 ± 1.32 ^c^ | 6.82 ± 1.38 ^a^ |
| Bitterness | 3.51 ± 1.48 ^d^ | 6.24 ± 1.04 ^b^ | 5.62 ± 1.38 ^c^ | 6.88 ± 1.20 ^a^ |
| Sweetness | 3.08 ± 2.11 ^d^ | 6.78 ± 1.55 ^b^ | 5.94 ± 1.56 ^c^ | 6.90 ± 1.30 ^a^ |
| Overall acceptability | 3.58 ± 1.34 ^d^ | 6.78 ± 1.27 ^b^ | 6.26 ± 1.14 ^c^ | 7.54 ± 1.16 ^a^ |
| Data are presented as mean ± standard deviation (SD).  Means within the same row followed by different superscript letters (a, b, c, ...) are significantly different (*p* < 0.05), as determined by one-way ANOVA with Tukey’s HSD post-hoc test.  Sensory liking and acceptance were evaluated using a 9-point hedonic scale.  Strain abbreviations: LP2070 = *L. plantarum* TISTR 2070 and SB745 = *S. boulardii* CNCM I-745. | | | | |

**Table S2.** Sensory evaluation of the fermented coffee cherry pulp beverages

| Color parameters | L* | a* | b* |
| --- | --- | --- | --- |
| Unfermented | 9.38 ± 0.03 ^A^ | 32.04 ± 0.09 ^D^ | 11.57 ± 0.10 ^D^ |
| LP2070 | 8.62 ± 0.03 ^B^ | 33.77 ± 0.07 ^B^ | 14.64 ± 0.08 ^B^ |
| SB745 | 7.74 ± 0.04 ^C^ | 32.90 ± 0.12 ^C^ | 13.14 ± 0.16 ^C^ |
| Co-culture | 6.92 ± 0.03 ^D^ | 34.50 ± 0.07 ^A^ | 15.94 ± 0.15 ^A^ |
| Values are expressed as mean ± SD of n=3 independent experiments.  Means within the same column followed by different superscript letters (A, B, C, ...) are significantly different (*p* < 0.05), as determined by one-way ANOVA with Tukey’s HSD  post-hoc test.  Strain abbreviations: LP2070 = *L. plantarum* TISTR 2070 and SB745 = *S. boulardii*  CNCM I-745. | | | |

**Table S3.** Color parameters (CIE L*a*b*) of coffee cherry pulp beverages.
