# Supplementary material for: Enhanced sensory, antioxidant, and non-toxic anti-diabetes beverage development via co-culture fermentation of Lactiplantibacillus plantarum and Saccharomyces boulardii in coffee cherry pulp extracts
Source: Food Chem X. 2026 Jan 12;33:103524. doi: 10.1016/j.fochx.2026.103524 (PMC12853062; doi:10.1016/j.fochx.2026.103524)
Supplement: Supplementary file 2 — Supplementary material 2 [file mmc2.pdf]

COA No. 056/68 CMUREC No. 68/043

หนังสือรับรองการพิจารณาจริยธรรมโครงการวิจัย
(Certificate of Approval)

ชื่อโครงการ: การเพิ่มคุณสมบัติต้านอนุมูลอิสระในสารสกัดเปลือกเชอร์รี่กาแฟ ผ่านกระบวนการหมักร่วมกับแบคทีเรียกรดแล็กติกและยีสต์ เพื่อพัฒนาผลิตภัณฑ์เครื่องดื่มเพื่อสุขภาพ

Project title: Enhancing Antioxidant Properties in Coffee Cherry Pulp Extract Through Optimized Co-Culture Fermentation for Functional Beverage Development

ผู้วิจัยหลัก: ศุภณัฐ โปธิมอย

Principal Investigator: Supanut Pothimoi

สังกัดหน่วยงาน: วิทยาลัยพหุวิทยาการและสหวิทยาการ มหาวิทยาลัยเชียงใหม่

Affiliation: Multidisciplinary and Interdisciplinary School, Chiang Mai University

วิธีการทบทวน (Reviewed Method): การพิจารณาแบบลดขั้นตอน (Expedited review)

เอกสารรับรอง:
1. โครงร่างการวิจัย
2. เอกสารชี้แจงผู้เข้าร่วมการวิจัย
3. หนังสือแสดงความสมัครใจในการเข้าร่วมการวิจัยฯ
4. เครื่องมือที่ใช้ในการเก็บข้อมูล
5. เอกสารประชาสัมพันธ์เชิญชวนผู้เข้าร่วมการวิจัย
6. ประวัติผู้วิจัย

Approved Documents:
1. Research Proposal
2. Participant Information Sheet
3. Informed Consent Form
4. Research tool for data collection
5. Research Recruitment Flyer
6. Researcher CV

คณะกรรมการจริยธรรมการวิจัยในคน มหาวิทยาลัยเชียงใหม่ ขอรับรองว่าโครงการวิจัยดังกล่าวข้างต้นได้รับการรับรองการพิจารณาจริยธรรมโครงการวิจัย ตามแนวทางหลักจริยธรรมการวิจัยในคนที่เป็นมาตรฐานสากล ได้แก่ ประกาศเฮลซิงกิ แนวทางการปฏิบัติการวิจัยทางคลินิกที่ดี และรายงานเบลมองต์

This is to certify that Chiang Mai University Research Ethics Committee has reviewed and approved the above research protocol based on international guidelines for human research protection including the Declaration of Helsinki, International Conference on Harmonization in Good Clinical Practice (ICH-GCP) and The Belmont Report.

ลงนาม (Signed)..

(รองศาสตราจารย์ ดร.ธันยวัฒน์ รัตนสัค)
ประธานคณะกรรมการจริยธรรมการวิจัยในคน มหาวิทยาลัยเชียงใหม่
(Associate Professor Dr.Thanyawat Rattanasak)
Chairperson, Chiang Mai University Research Ethics Committee

วันที่รับรองการพิจารณาจริยธรรม: 11 กุมภาพันธ์ 2568
Date of approval: 11 February 2025

วันหมดอายุ: 10 กุมภาพันธ์ 2569
Date of expiration: 10 February 2026
